# Complex CDKL5 translational regulation and its potential role in CDKL5 deficiency disorder

**DOI:** 10.3389/fncel.2023.1231493

**Published:** 2023-10-30

**Authors:** Valeria Ruggiero, Claudio Fagioli, Stefano de Pretis, Valerio Di Carlo, Nicoletta Landsberger, Daniele Zacchetti

**Affiliations:** ^1^Vita-Salute San Raffaele University, Milan, Italy; ^2^Division of Neuroscience, IRCCS San Raffaele Scientific Institute, Milan, Italy; ^3^Department of Medical Biotechnology and Translational Medicine, University of Milan, Segrate, Italy

**Keywords:** CDKL5, translation initiation, 5′UTR, CDKL5 deficiency disorder, IRES

## Abstract

CDKL5 is a kinase with relevant functions in correct neuronal development and in the shaping of synapses. A decrease in its expression or activity leads to a severe neurodevelopmental condition known as CDKL5 deficiency disorder (CDD). CDD arises from CDKL5 mutations that lie in the coding region of the gene. However, the identification of a SNP in the CDKL5 5′UTR in a patient with symptoms consistent with CDD, together with the complexity of the CDKL5 transcript leader, points toward a relevant translational regulation of CDKL5 expression with important consequences in physiological processes as well as in the pathogenesis of CDD. We performed a bioinformatics and molecular analysis of the 5'UTR of CDKL5 to identify translational regulatory features. We propose an important role for structural cis-acting elements, with the involvement of the eukaryotic translational initiation factor eIF4B. By evaluating both cap-dependent and cap-independent translation initiation, we suggest the presence of an IRES supporting the translation of CDKL5 mRNA and propose a pathogenic effect of the C>T -189 SNP in decreasing the translation of the downstream protein.

## 1. Introduction

Translational control of gene expression is an established regulatory step, downstream of transcription, to regulate the level of specific proteins with high speed and precise localization (Hershey et al., 2019; Skariah and Todd, 2021). Eukaryotic cells exploit several tricks to obtain this regulation, some of which occur during the first steps of translation modulating the efficiency of the overall process (Hershey et al., 2019). Indeed, in the context of translational initiation,the transcripts. High there are many mechanisms involving cis-acting regulatory elements within the sequence of the 5′UTR of the transcripts. High-structured 5′UTRs can both decrease or enhance translation efficiency, working either as barriers during the canonical cap-dependent translation initiation or as platforms for the direct recruitment of the small ribosomal subunit, such as in the Internal Ribosomal Entry Site (IRES)-mediated non-canonical initiation mechanism (Leppek et al., 2018). Upstream start codons placed in the 5′UTR similarly work as inhibitory elements of translation from the main start codon although they can even act as helpers of its translation (Hinnebusch et al., 2016). Regulation can include the interaction with trans-acting factors, such as the eukaryotic initiation factors (eIFs) and other accessory proteins (Hinnebusch et al., 2016; Leppek et al., 2018) or RNA molecules, such as microRNA (Gu et al., 2014) and lnRNA (Verheyden et al., 2018). In particular, the dynamic interplay between eIFs and the cis-acting regulatory elements contained in the 5′UTR sequences includes the modulation of the phosphorylation status of eIF2 causing the skipping of inhibitory upstream start codons (uAUGs and others) (Hinnebusch, 1994; de Haro et al., 1996; Hinnebusch et al., 2016) and the involvement of the DEAD-Box helicase eIF4A in the unwinding of regulatory secondary structures (Parsyan et al., 2011; Andreou and Klostermeier, 2013). It is worth to mention that the action of these factors is often mediated by other proteins or molecules that dynamically interpret the cellular requests to optimize the rate of translation. For example, the obligatory function of eIF4A is fine-tuned by its preferential helper eI4FB, the phosphorylation of which translate the tissue-specific signals produced by various signaling pathways in differential unwinding efficiency (Chen et al., 2016; Bettegazzi et al., 2017, 2021). The importance of 5′UTR as an important player in the expression of the proteins is also demonstrated by the fact that many variants found in this sequence can cause an alteration in the translation efficiency of proteins linked to monogenic disorders, such as the complete androgen insensitivity syndrome (CAIS), thrombocytopenia 2 (THC2), and fragile X syndrome (FXS) (Hornig et al., 2016; Marconi et al., 2017; Crowley et al., 2019). Moreover, a SNP in the c-Myc 5′UTR has been found to cause the overexpression of c-Myc in multiple myeloma (Chappell et al., 2000; Shi et al., 2016), and SNPs in the cis-acting regulatory motifs in the transcript leaders of connexin 32 and VEGFA has been linked to the development of two severe neurodegenerative disorders, the Charcot Marie Tooth disease and the amyotrophic lateral sclerosis (Hudder and Werner, 2000; Lambrechts et al., 2003). The emerging link between altered translational control of protein expression and neurodevelopmental and neurodegenerative human disorders (Jishi et al., 2021) is not surprising since translational regulation is particularly adopted by neurons, that need a very tight, sophisticated control of protein synthesis. In fact, their morphology and function require a highly dynamic and local control of protein expression since synapses are continuously tuned according to their activity that occurs far away from the nucleus (Holt et al., 2019).

Cyclin-dependent kinase-like 5 (CDKL5), a protein enriched in neurons and involved in the regulation of their maturation and synaptic plasticity (Barbiero et al., 2017), has been reported to be under translational control in neurons since it is locally translated at the post-synaptic site in an activity dependent manner (La Montanara et al., 2015). CDKL5 transcript is characterized by a first exon (Ex1) containing only untranslated nucleotides, followed by a second exon (Ex2) containing a 162-long distal untranslated region (untranslated exon 2, UTex2) and a proximal coding region of the gene in which there is the start codon, at position 18.507.097 of the human X chromosome (Hector et al., 2016). The combination of the two untranslated regions, Ex1 and UTex2, forms the CDKL5 5′UTR. However, the CDKL5 gene can produce several transcript variants with various 5′UTR sequences differing according to the choice of an alternative Ex1 (Hector et al., 2016, 2017a,b). Little is known about the relevance and the expression levels of these 5′UTRs, as well as their functions. Interestingly, variants in the 5′UTR of CDKL5 have been identified in some patients affected by CDKL5 deficiency disorder (CDD), a severe loss-of-function monogenic neurodevelopmental syndrome characterized by cognitive disabilities, autistic-like features, refractory epilepsy, and hypotonia (Olson et al., 2019). Although most of the variants in the 5′UTR region induce splicing alternation and exon skipping (Nemos et al., 2009; Bahi-Buisson et al., 2010; Mei et al., 2010; Jähn et al., 2013), a SNP found in a patient with symptoms consistent with CDD (Evans et al., 2005) is not expected to influence neither transcription nor splicing. This leaves open the possibility of an interference with translation initiation, leading to a shortage of the corresponding protein product. In this work, we aimed to characterize the various CDKL5 5′UTRs to investigate their role in the modulation of translational efficiency and their potential relevance for CDD. We evaluated possible cis-acting regulatory elements as well as canonical and non-canonical translational mechanisms related to secondary structural motifs. According to our findings, we propose an unforeseen role for CDKL5 transcript leader in the regulation of the expression of the kinase as well as in a possible new CDD onset mechanism.

## 2. Materials and methods

### 2.1. Sequence conservation analysis

The sequences of the human variants of the transcript leader of CDKL5 were acquired from the GRCh38.p13 assembly of Ensembl (Howe et al., 2021) and published literature (Hector et al., 2016). Sequence conservation analysis was performed starting from a nBLAST search (Sayers et al., 2021) in the nucleotide database. The search parameters were optimized for non-coding regions, setting a word-size of seven bases (BlastN algorithm), and an expected threshold (*E*) of 0.1. Match/mismatch scores of 1/-2 and gap costs of 1 for existence and 1 for extension were selected. nBLAST search in the EST database was conducted with the same parameters. Multiple sequence alignment of the chosen 26 sequences (Supplementary material 2) was performed using Clustal-omega (Sievers and Higgins, 2014) and visualized through Consurf (Ashkenazy et al., 2016). In Consurf, we selected the calculation method maximum likelihood (ML) and the evolutionary substitution model HKY85 (Hasegawa et al., 1985). Consurf scores for each nucleotide indicates the conservation at the given position (1 = not conserved; 9 = very conserved). These scores were analyzed through the Prism software (GraphPad Software), applying a sliding window on the arithmetical means of five nucleotide positions to obtain the conservation profile of the sequence of interest.

### 2.2. Cis-acting regulatory elements predictions

The prediction of the translation initiation start sites was conducted using two tools with different algorithms: Netstart (Pedersen and Nielsen, 1997) and TIS Miner (Liu et al., 2005). The calculation of the GC content of the sequences was performed trough the mathematical formula:


(1) $$\begin{array}{l} \left[\left(G+C\right)/\left(G+C+A+T\right)\right]*100 \end{array}$$

and expressed as percentage. Watson and Crick secondary structure prediction was performed using RNAfold (Mathews et al., 1999; Hofacker, 2003). The predicted structures were interpreted following the reported guidelines (Gruber et al., 2008): (i) the stability of the structures predicted trough the positional entropy; (ii) the ensemble diversity (ED), as a measurement of the variability of the possible secondary structures folded from the same sequence; (iii) the distance between the GMFE the GCE indices, as a measurement of the reliability of the predicted MFE structure. RNA structures were visualized through forna (Kerpedjiev et al., 2015). The QGRS Mapper program (Kikin et al., 2006) was used to predict G-quadruplex motifs.

### 2.3. Cell culture

SHSY-5Y cells was cultured in RPMI 1640 media (Gibco) supplemented with 10% Fetal Clone III (Thermofisher Scientific), 1% penicillin/streptomycin (Thermofisher Scientific), and 1% non-essential amino acids (Cyagen). After trypsinization with 0.25% tripsyn/EDTA, Sigma/Aldrich cells were plated in six-well multiwell plates and used for transfection.

### 2.4. Tissue samples preparation

CD-1 wild-type mice were treated following the European Community Council Directive 2010/63/UE for the correct care and use of experimental animals. When the experimental animals reached the 30th postnatal day, they were dissected to take cortices. RNA Later Buffer (Invitrogen) was used to preserve the quality of collected samples at −20°C before RNA extraction.

### 2.5. RNA extraction and retrotranscription

Cells cultures were washed with PBS and lysed with PureZOL (BioRad) 5 min at room temperature directly on plates (300 μl/well). Lysate was collected, 60 μl/well of chloroform (Merck) was added and total RNA was prepared according to the standard PureZOL protocol. The same protocol was adopted for mice sample, except for the addition of a previous step, in which mouse cortices were homogenized in 500 μl of PureZOL, using the Tissues Lyser (Qiagen). Once purified, the RNA samples were quantified with Nanodrop (ThermoFisher) and retrotranscribed with the RT2 Easy First Strand Kit (Qiagen).

### 2.6. RT-PCR

We designed the PCR primers by means of NetPrimer (PREMIER Biosoft), and we checked their quality through in-silico PCR (UCS Genome Browser). The list of primers used for the amplification of murine samples is as follows:

- mCDKL5 TOP1 5′ TACTTGTCGCTGCCGCTAGGGA 3′
- mCDKL5 TOP2 5′GCTCCGGCGAGAGGGCGGGG 3′
- mCDKL5 TOP3 5′GCAGACGGGGGCGGTGCGA 3′
- mCDKL5 REVERSE 5′TAATGTCCCAACGAAGAAATTCTC 3′
- mGAPDH FORWARD 5′ AGGTCGGTGTGAACGGATTTG 3′
- mGAPDH REVERSE 5′TGTAGACCATGTAGTTGAGGTCA 3′

The list of primers designed for the amplification of SHSY5Y samples is as follows:

- hCDKL5 TOP1 5′TAGTTGTCTCTGCCGCTGGGGA 3′
- hCDKL5 TOP2 5′CTTCTGCTAGAGGGCGGGG 3′
- hCDKL5 TOP3 5′GCTGGGGCGGGGCAGTTAG 3′
- hCDKL5 REVERSE 5′CACTGGTTGGTGGGAACTTTCAC 3′
- hGAPDH FORWARD 5′GTCTCCTCTGACTTCAACAGCG 3′
- hGAPDH REVERSE 5′ACCACCCTGTTGCTGTAGCCAA 3′

PCR reaction was carried out by Taq Polymerase (ThermoFisher) in 50 μl starting from 100 ng of cDNA with annealing at 63°C and extension at 70°C. The amplification products, collected at the 27th cycle for GAPDH and the 35th cycle for CDKL5, were loaded on a 2% agarose gel and visualized by a Gel Doc XR (BioRad).

### 2.7. eIF4B gene silencing

Small interfering RNA transfections were performed using Lipofectamine 3000 Reagent (Thermo Fisher) with 100 pmol/ml of siRNA following the instructions. eIF4B siRNA was purchased by Dharmacon (ON-TARGETplus Human eIF4B siRNA, SMARTPool format). SHSY-5Y cells were transfected at 80% of confluency and then incubated for 72 h at 37°C. After this period, we performed cell lysis using RIPA Buffer (50 mM Tris/HCl pH 8, 150 mM NaCl, 1% NP40, 0.1% SDS, 0.5% sodium deoxycholate. In the study, 5 mM EDTA and protease inhibitor mix (CLAP: chymostatin, leupeptin, aprotinin, PMSF). Detection of proteins was performed by SDS-PAGE followed by Western blot analysis. The primary antibodies were αtubulin (Cell Signaling, 3873); CDKL5 (Santa Cruz Biotech., Sc-376314); eIF4B (Cell Signaling, 3592); and XIAP (Cell Signaling, 14334). Detection and quantification of chemiluminescence signals were performed by SuperSignal West Pico substrate (ThermoFisher) using the Chemidoc Imaging System and the ImageLab software (BioRad).

### 2.8. Reporter constructs for luciferases assays

We purchased the pBRm2L plasmids with the inserts of interest (5′UTRs sequences) from Synbio Technology after providing the original plasmid (De Pietri Tonelli et al., 2004).

- The plasmid pBRm2LEx1205 contains the CDKL5 5′UTR reported as variant 205 on Ensembl (RefSeq *NM*_001323289.2), the sequence of which is AGTTGTCTCT GCCGCTGGGG AAGGTAAAGC GGCGACGGCG TCCTCAGGAG CTGTGGGGTC CCCTGCTAGA AGTGGGGGAC TCGGCGGGGG AGTCATTTAA TACTTCATGA TTAGAACAAA TATGTGAAAG TTCCCACCAA CCAGTGAGAA TTTCTTCCTT CAGACGGTTT TGGATCTTAC TGCACAGCTT TCTGAGAAGT TCTTTTGGTG CCATGTTTTG TGGCTTGCAT CAAAAGAGGA GTTTGTCTTC.
- The plasmid pBRm2LEx1202up contains the complete 5′UTR sequence carrying the Ex1 202 plus the 10 upstream nucleotides found by RT-PCR in human and murine transcriptomes with the sequence: GCTTCTGCTA GAGGGCGGGG CCGGAGGTTT CGATTAGTTG TCTCTGCCGC TGGGGAAGGT AAAGCGGCGA CGGCGTCCTC AGGAGCTGTG GGGTCCCCTG CTAGAAGTGG GGGACTCGGC GGGGGAGTCA TTTAATACTT CATGATTAGA ACAAATATGT GAAAGTTCCC ACCAACCAGT GAGAATTTCT TCCTTCAGAC GGTTTTGGAT CTTACTGCAC AGCTTTCTGA GAAGTTCTTT TGGTGCCATG TTTTGTGGCT TGCATCAAAA GAGGAGTTTG TCTTC.
- The plasmid pBRm2LUTex2 contains only the UTex2 region of the CDKL5 5′UTR with the sequence GGAGTCATTT AATACTTCAT GATTAGAACA AATATGTGAA AGTTCCCACC AACCAGTGAG AATTTCTTCC TTCAGACGGT TTTGGATCTT ACTGCACAGC TTTCTGAGAA GTTCTTTTGG TGCCATGTTT TGTGGCTTGC ATCAAAAGAG GAGTTTGTCT TC.
- The plasmid pBRm2LEVA contains the complete 5′UTR of the variant 202up with the rs786204994 SNP (Evans et al., 2005) at position -189 (C>T).

The CDKL5 inserts of pBRm2L were amplified by PCR. SalI and NcoI restriction sites were added at the end of the primers. After digestion of the amplified products with the two restriction enzymes (NEB) for 2 h at 37°C, the fragments were inserted by ligation with the Quick Ligation Kit (NEB) in the pBATmod2 plasmid (De Pietri Tonelli et al., 2003) digested with the same enzymes and pretreated with rSAP (NEB). The new plasmids were verified by LightRun sequencing service (Eurofins Genomics) and named pBATmod2Ex1205, pBATmod2Ex202up, pBATmod2UTex2, and pBATmod2EVA.

### 2.9. Reporter transfection and luciferase assay

SHSY-5Y cells at 70%–80% of confluency on 24-well plates were first infected with the Modified Vaccinia virus Ankara (MVA), carrying the T7 RNA polymerase gene, and then transfected (according to De Pietri Tonelli et al., 2003). For DNA transfection, we used 500 ng of vector using Lipofectamine 3000 (ThermoFisher) following the manufacturer protocol. Cells were incubated at 37°C for 5 h, washed twice with PBS, lysed with passive lysis buffer and processed to detect the signals from FLuc and RLuc using the Dual-Luciferase Reporter Assay System (Promega) on a GloMax reader (Promega). We calculated the translation efficiency of FLuc as the ratio between FLuc and RLuc signals. All the measurements were repeated twice for each sample (20 μl of lysates) from at least three independent experiments performed in triplicate.

### 2.10. Transcription start site bioinformatic analysis

We performed transcription start site (TSS) peak quantification analysis on CAGE libraries of the following human tissues: adult brain, adult kidney, adult testis, adult spleen, adult lung, adult heart, adult liver, and fetal brain, made available by the FANTOM5 consortium. CAGE tags were analyzed using CAGEr package, freely distributed by R/Bioconductor (Haberle et al., 2015). Normalization of the analysis was performed in accordance with Balwierz et al. (2009). The same analysis was performed also on murine CAGE libraries from various tissues (cortex, lung, liver, heart, testis, and kidney) and developmental stages (embryonic, neonatal, and adult).

## 3. Results

### 3.1. Evaluation of known CDKL5 5′UTR variants and sequence conservation analysis

**Figure 1:**
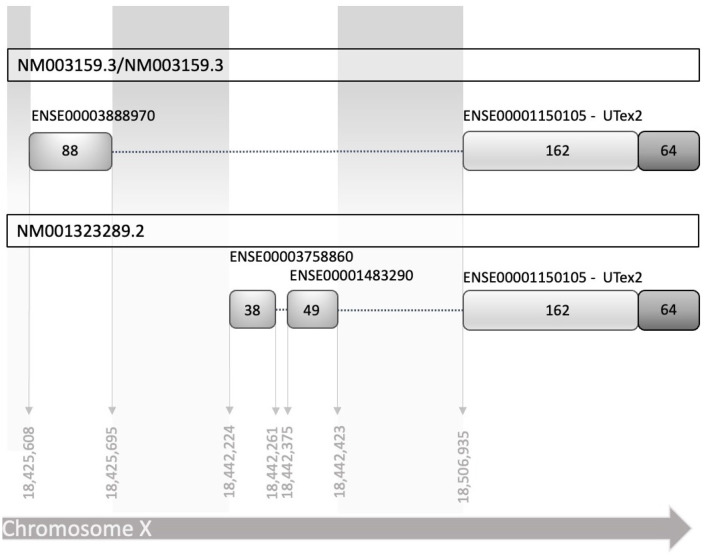
Schematic diagram of human reference sequences of the CDKL5 5′UTR (NM003159.3 NM003159.3, and NM001323289.2). The two transcript leaders share a common part of 162 nucleotides in exon 2, referred in the text as UTex2, while differ in the upstream part, in which there are at least two alternative TSS, with two different exons and an additional splicing mechanism. Exons are indicated with the stable identifiers reported in Ensembl, with the number of nucleotides that compose them expressed as absolute number inside the boxes representing the exons. Dotted lines linking the exons represent the introns (not in scale).

To establish the conditions to investigate the translational regulation of CDKL5, we listed all the known 5′UTR and built a comprehensive catalog of all possible variants (Supplementary material 1). Consistent with the CDKL5 transcript leaders reported in the NCBI reference sequences database (NM003159.3/NM003159.3 and NM001323289.2), all the analyzed sequences have a common organization, sharing the sequence that is proximal to the main translational start site, consisting of 162 nucleotides in the exon 2 (UTex2), and differing at the distal 5′ region (Figure 1). We considered the 16 alternative first exons reported in Table 1 arising from various transcription start sites (TSSs), as previously claimed in other studies (Hector et al., 2016). A part of these sequences is only predicted (see Ensembl), whereas the experimentally defined first exons (Hector et al., 2016, 2017b) are not included in any reviewed databases. Moreover, most of the exons differ for the length at the 5′ end, a feature often associated with technical limitations (i.e., degradation of the mRNA or interrupted retrotranscription due to tight structures) in defining the first nucleotides of a transcript.

**Table 1:** List of all the CDKL5 human alternative first exons found in Ensembl and in literature (Hector et al., 2016).

**ID**	**Start**	**End**	**Annotation**
Ex1 202	18425583	18425695	ENSE00001356011
Ex1 205	18425608	18425695	ENSE00003888970, Ex1 2013, Ex1 HecA
Ex1 HecA1	18426069	18426401	–
Ex1 207	1844228	18442423	ENSE00003758990
Ex1 209	18426198	18426401	ENSE00003795706
Ex1 HecB1	18426691	18426919	–
Ex1 211	18426713	18426919	ENSE00003798952
Ex1 HecB	18426876	18426919	–
Ex1 201a	18442188	18442261	ENSE00001483291
Ex1 208a	18442213	18442423	ENSE00003757043
Ex1 204a	18442224	18442261	ENSE00003758860, Ex1 HecC
Ex1 203	18442275	18442390	ENSE00003759860, Ex1 HecF
Ex1 204b	18442375	18442423	ENSE00001483290, Ex1 201b, Ex1 HecD
Ex1 208b	18443819	18443959	ENSE00003757121, Ex1 HecE1
Ex1 HecE	18443839	18443959	–
Ex1 204c	18457483	18457534	ENSE00003760056

The sequences are identified by code (ID), using the Ensembl reference nomenclature when the sequences are reported in the Ensembl transcript variants, or using the prefix Hec when uniquely detected in the paper by Hector et al. (2016). The 5′ (Start) and the 3′ (End) exonic limits are indicated in columns. Other possible IDs (including the NCBI reference sequences NCBI) for each variant are reported in the annotation column. The reference genome was hg38.

**Figure 2:**
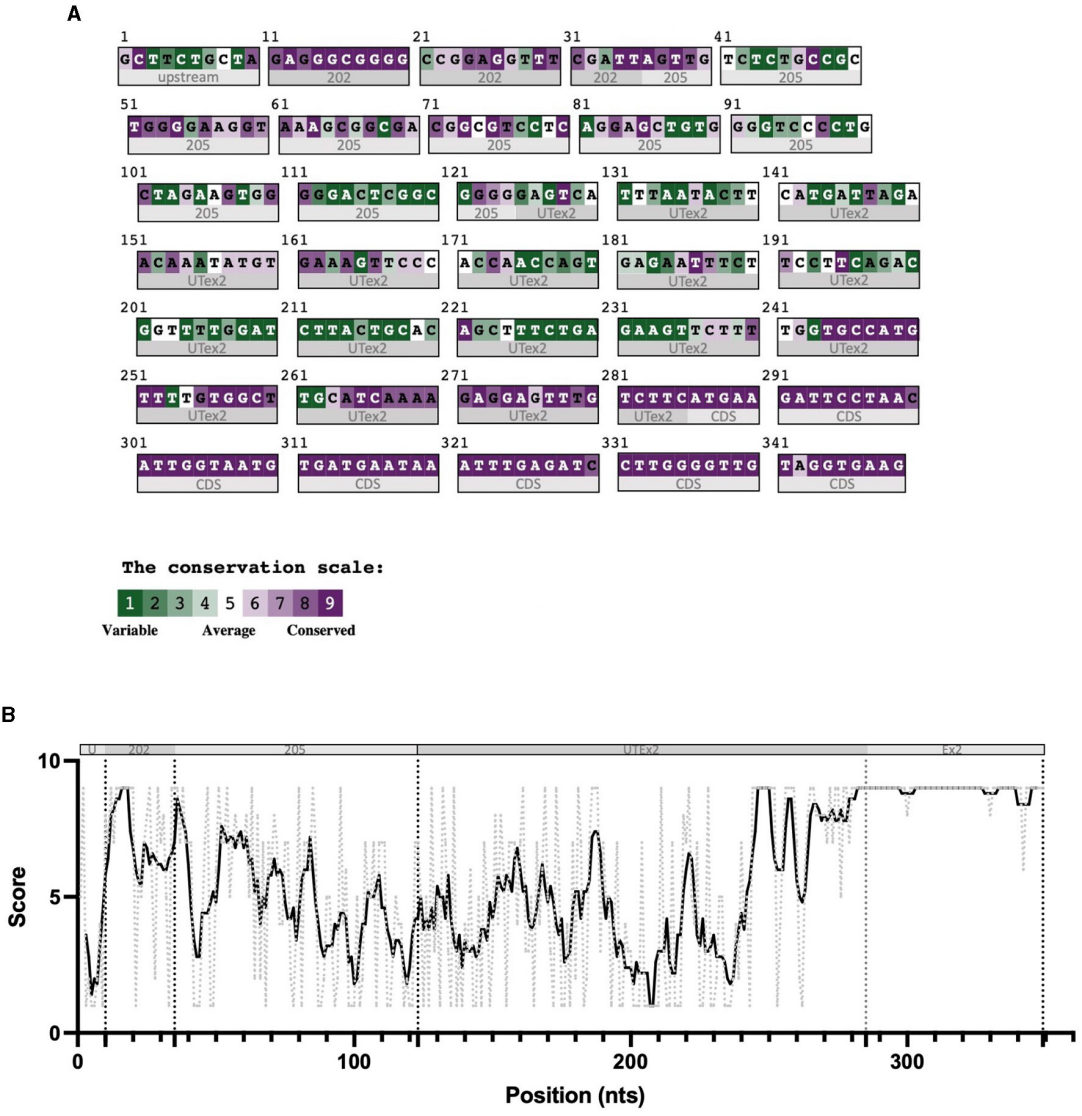
Color-coded summary of the alignment of 64 homologous sequences to the human query 5′UTR 202 and as score versus position graph. The sequences used to build the alignment at the base of these representations were obtained from an nBLAST search in the nucleotides database, using the human sequence as query. This search returned 64 mammalian homologous sequences, aligned through Clustal-omega. Subregions of the sequence are indicated below the graph and under the color-coded alignment as follows. **(A)** In panel A, 202 indicates the exclusive nucleotides of the 202 exon 1 sequences, 205 indicates the nucleotides of the 205 exon 1 sequence common to the 202 exon 1, and UTex2 refers to the untranslated part and Ex2 the coding region of the exon 2. **(B)** The various 5'UTR's sequence regions are divided by dotted lines (U = upstream the 202 TSS; 202 = exclusive part of the 202 TV; 205 = first exon of 205 TV; UTex2 = untranslated region of exon 2; Ex2 = translated region of exon 2). The black bold line represents the conservation trend of the sequence, which is calculated as the moving average of position scores (indicated on the *y* axis for each position) over the entire sequence, using a window size of five nucleotides. The conservation analysis reveals the high conservation of the nucleotides at the 5′ part of the first exon.

**Figure 3:**
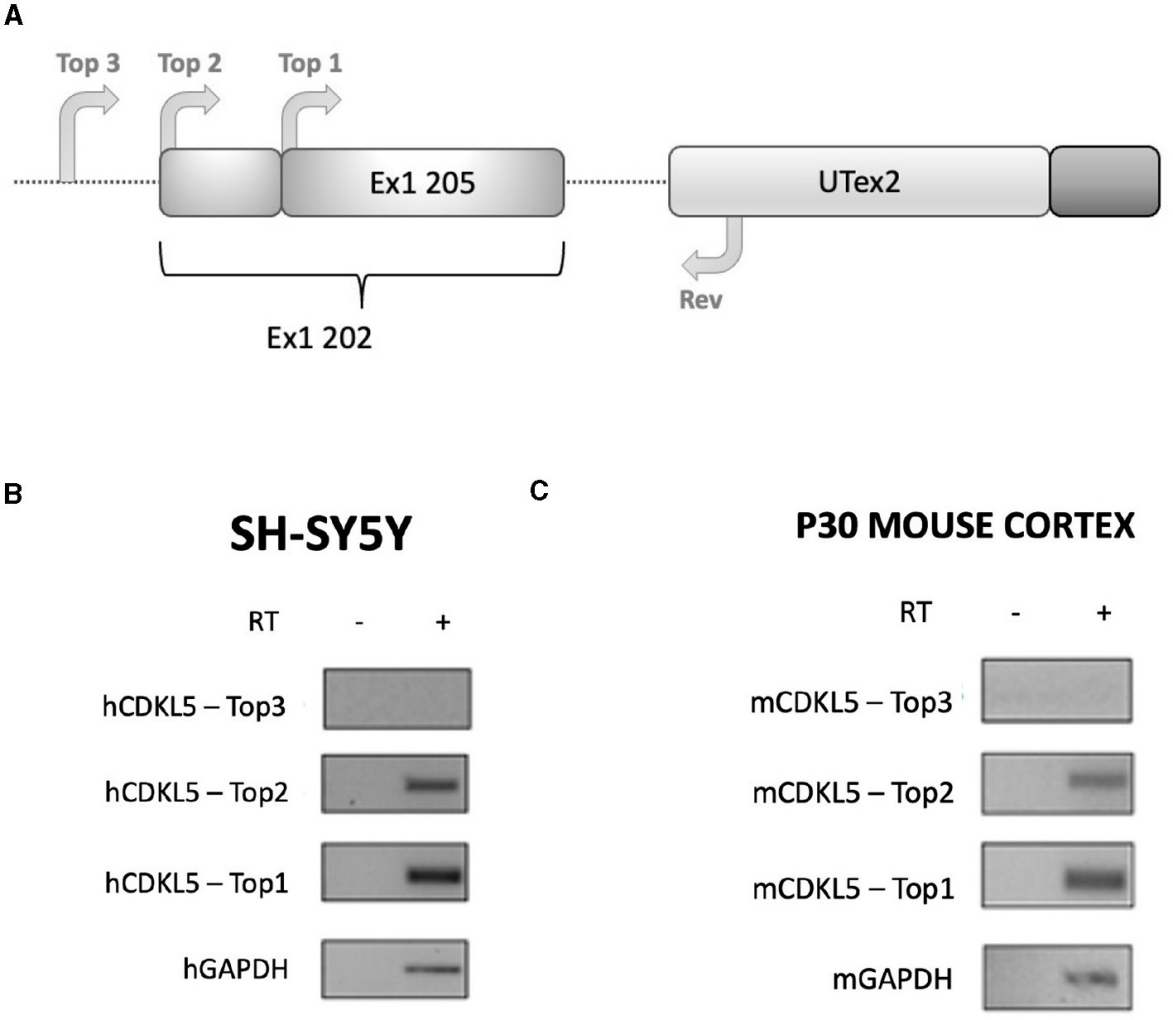
RT-PCR analysis assessing the existence of the sequence Ex1 202 in human and murine transcriptomes. **(A)** Experimental design diagram in which the positions of the primers are schematically reported on the CDKL5 5′UTR sequence. Three forward primers (Top1, Top2, and Top3) are employed in the analysis to assess the length of the sequence, while a common reverse primer (Rev) is placed on UTex2. Top1 is set on the 205 sequence, Top2 was set on the reported 202 TSS, and Top3 was set 11 nucleotides upstream the first nucleotides of Top2. **(B)** Agarose gel electrophoresis of the PCR products obtained from SHSY-5Y cDNA. The Top1-Rev couple of primers returned the expected 202 signal. The Top2-Rev amplification product confirmed the presence of at least 10 upstream nucleotides from the 202 TSS. The Top3-Rev primers did not return any detectable amplification product. A non-retrotranscribed sample was used as control. GAPDH amplification was performed as technical control. **(C)** The same analysis performed in P30 wild-type murine cortices gave the same results.

For these reasons, we performed sequence conservation analysis to screen sequences in the predicted CDKL5 transcripts and to play a role in protein synthesis modulation, following the assumption that conserved untranslated sequences have a higher chance to retain a regulatory function (Hardison, 2000). Starting from UTex2, the sequence conservation analysis predicted that it is anciently conserved, with homology in more than 206 genomes, including mammals, reptiles, and birds (not shown). This result is strengthened by the returned hits in the expressed sequence tags (EST) database, confirming the UTex2 detection in at least one bird transcriptome (Lonchura striata domestica: DC278838.1). Concerning the first exons, almost all the sequences reported in Table 1 are not conserved. The first exon sequences that show a reliable conservation are the Ex1 205 (88 nucleotides) and Ex1 202 (113 nucleotides), with homologous hits in 64 mammalian species since marsupials, hinting the possible origin of these sequences in this infraclass (not shown; see also Supplementary material 2). By comparing Ex1 202 (i.e., 25 nucleotides + Ex1 205) and UTex2, it is interesting to note that the first exon is more conserved that UTex2 despite being evolutionary more recent, pointing toward a possible important function within the first exon sequence in mammals (Figures 2A, B). Interestingly, Ex1 202 and Ex 205 were the only ones to be confirmed by a search in the EST database, where both returned homology also in non-primate transcriptomes. The sequence Ex1 202 was found in four EST libraries from various primates and rodents. Notably, one of this returned a record, belonging to the marmoset monkey (Callithrix jacchus: HX595850.1), that exceeds the 5′ limit of the human query, suggesting the existence of a longer version of the Ex1 202 also in humans. To verify this possibility, we performed a PCR-based approach on cDNA obtained from SHSY5Y cells and p30 murine cortices (Figure 3A). The analysis detected the complete sequence of Ex1 202 but also confirmed the presence of additional nucleotides upstream the reported TSS of the 202 variant (+10 nucleotides, Figure 3B). This longer version of the Ex1 202 was named Ex1 202up.

### 3.2. 5′UTR of CDKL5 contains predicted structured cis-acting regulatory elements

**Figure 4:**
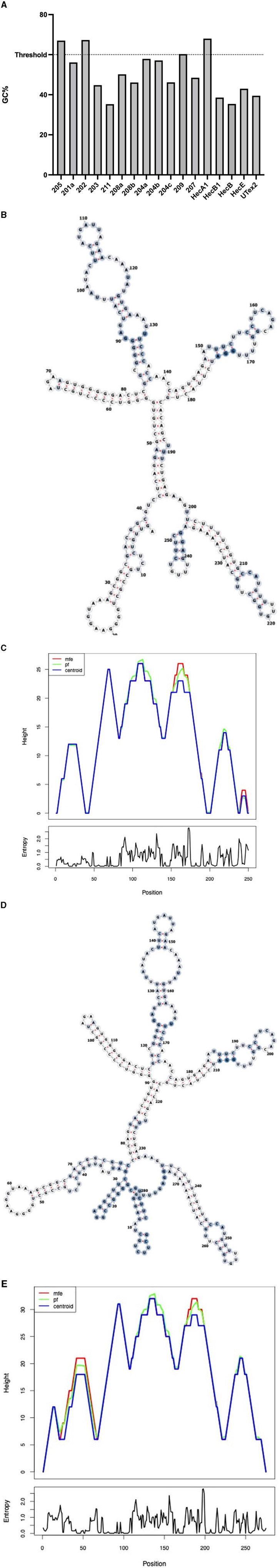
Structural predictions of the 5′UTRs carrying the 205 and 202 first exons. **(A)** Percentage of GC bases in the exonic sequences of the CDKL5 transcript leader (UTex2 included). Most of the sequences do not reach the 60% threshold (Davuluri et al., 2000) except for the alternative 205, 202, and HecA1 first exons. **(B)** MFE structure of the complete 205 sequence (Ex1 205 + UTex2) is displayed through the forna software, allowing to visualize the positional entropy at each nucleotide sites (blue for high and white for low entropic positions). The structure appears well ordered and displays tight stem loops in its folding. **(C)** Mountain plot gives a graphical representation of the reliability of the predicted secondary structures as comparison of the result obtained with the three algorithms used by RNAfold (MFE, pf and centroid). Positional entropy plot of the 205 sequence provides a linear entropic profile that highlights the role of the first exon in the stability of the entire sequence. **(D)** MFE structure of the complete 202up sequence (10 nucleotides + Ex1 202 + UTex2) by forna is similar to the predicted structure of the 205 transcript leader, except that the beginning of the sequence shows higher disorder as indicated by the coloration (blue for high and white for low entropic positions). **(E)** Mountain plot returns the reliability of the predicted structures showing the discrepancy between the three algorithms at the beginning of the 202up sequence. Positional entropy plot of the 202up 5′UTR.

**Figure 5:**
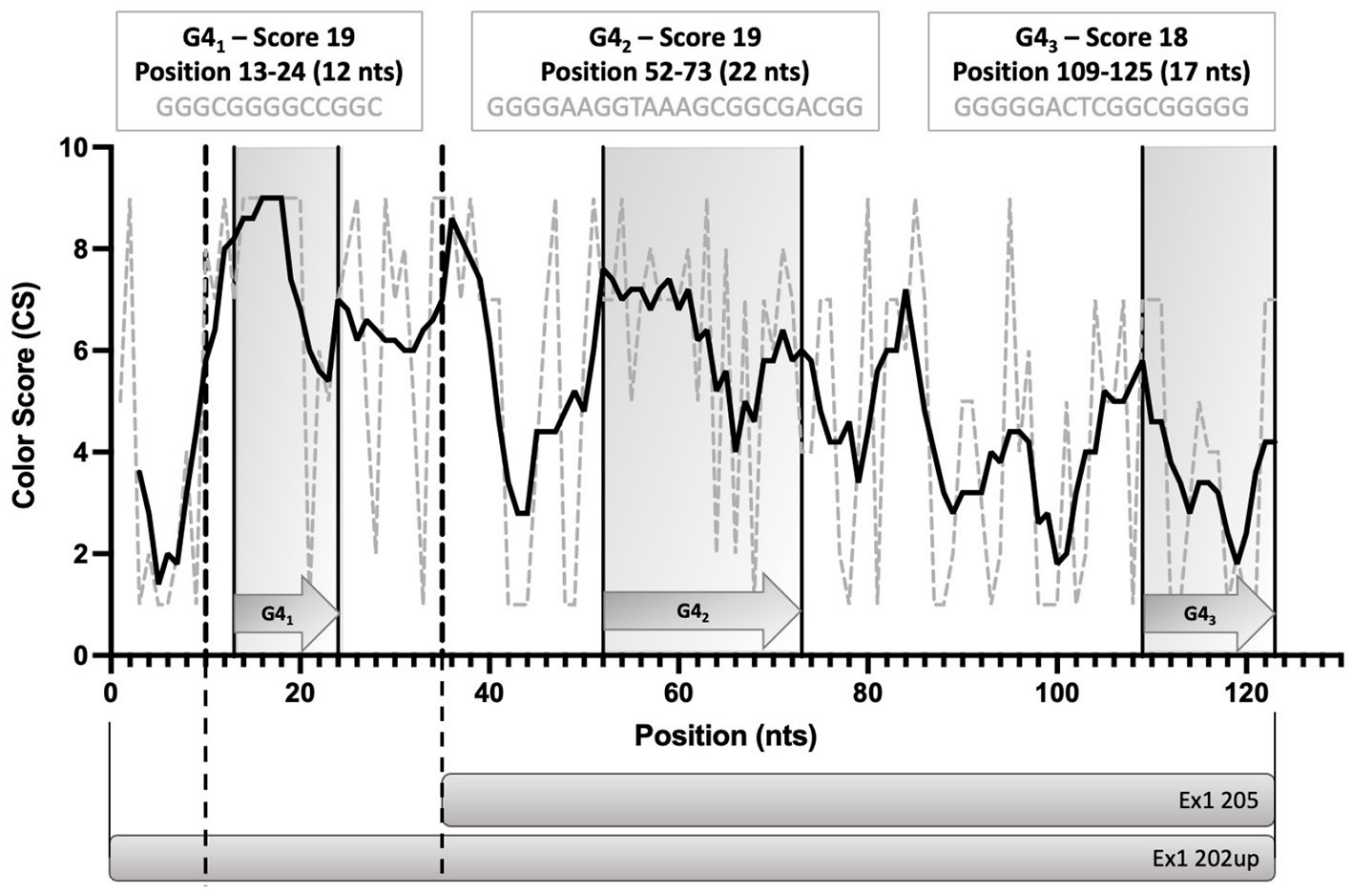
Schematic diagram of the position of the three predicted G-quadruplexes (*pG*4_1_, *pG*4_2_, and *pG*4_3_) in the 5′UTR variants 202up and 205. The length and the G-score of each of the predicted motifs is reported. The Consurf conservation analysis shows the different degree of conservation of the nucleotides involved in the pG4s. *pG*4_1_ is the most conserved motif, followed by *pG*4_2_. Dotted lines define the limits between the upstream 10 nucleotides of Ex1 202up, the Ex1 202 and the Ex 205 sequences. Straight lines delimitating gray boxes indicate the region in which pG4s lie. The prediction was performed using QGRS Mapper.

To evaluate the presence of cis-acting regulatory elements in the 5′UTR of CDKL5, we performed a predictive analysis on all the CDKL5 transcript leaders. We did not obtain any significant prediction of upstream start sites using two different algorithms (Netstart, Pedersen and Nielsen, 1997; TIS Miner; Liu et al., 2005, Supplementary Table S1). We then focused on possible regulatory secondary structures that might play a role in the modulation of translation initiation rate (Hinnebusch et al., 2016; Leppek et al., 2018). First, we calculated the GC percentage of the exonic sequences. Our analysis highlighted that the majority of the first exons and the common UTex2 did not have a particularly high GC percentage, while the conserved 205 and 202 sequences, as well as HecA1, exceed the conventional threshold of 60% of GC nucleotides (Figure 4A). This threshold is commonly associated to the presence of regulatory secondary structures (Davuluri et al., 2000). In line with this assumption, the global estimated Watson and Crick folding of all the possible CDKL5 5′UTRs returned disorganized and disordered prediction, except for the 205 and 202 variants, as shown by positional entropy (Table 2). These two sequences are characterized by a negative ΔGMFE of −91.6 and −101.4 kcal/mol, respectively. These values, and the corresponding predicted structures, can be considered highly reliable according to the guidelines of RNAfold since GCE is comparable to ΔGMFE and the Ensemble Diversity is relatively small (Gruber et al., 2008) (Table 2). Moreover, also the MFEden normalization (Trotta, 2014) that considers the length of the sequence confirms the reliability of the prediction (Supplementary Table S2). This analysis strengthened the selection of 202 and 205 sequences as interesting from a functional viewpoint. In the 205 5′UTR sequence, the 88 nucleotides composing the first exon give the greater contribution to the stability of the structure, involving various stem-loops, some of which formed together with the UTex2 nucleotides, with very low positional entropy (Figures 4B, C). The longer variants 202 and 202up returned, as expected, a very similar structure. However, the presence of the 25 (or 35 in the case of 202up) upstream nucleotides before the putative 205 TSS are characterized by a localized higher entropy level (Figures 4D, E). Since this entropy level profile in a G-rich region suggests the presence of G-quadruplexes, other secondary structures involved in translational regulation (Zhang et al., 2011; Bugaut et al., 2012; Takahashi and Sugimoto, 2021), we performed a G-quadruplex prediction on these sequences (as well as on all the reported exonic sequences of the CDKL5 transcript leader). The prediction returned the presence of three distinct motifs (namely, *pG*4_1_, *pG*4_2_, and *pG*4_3_) on the sequence Ex1 202up. While *pG*4_2_ and *pG*4_3_ are shared with the 205 sequence, *pG*4_1_, the most conserved of the three, lies in the exclusive 35 nucleotides of the variant 202up (nucleotides 13–24), hinting a possible functional difference between the two alternative first exons (Figure 5). From these bioinformatic analyses, the conserved and structured 5′UTR variants 205 and 202 (or 202up) appear to be the transcript leaders with the highest probability to play a role in the translational control of CDKL5.

**Table 2:** Data obtained from the secondary structure predictions performed using RNAfold.

**ID**	**MFE (Kcal/mol)**	**CE (Kcal/mol)**	**ED**
205	−91.6	−90.2	33.41
202	−101.4	−101.2	38.69
HecA1	−170.7	−141.6	146.08
207	−98.9	−105.1	68.9
209	−115.3	−79.1	106.55
HecB1	−99.2	−69.7	120.3
211	−93.4	−68.8	100.08
HecB	−55.1	−55.1	17.98
201	−88.8	−86	56.17
208	−148.9	−113.3	144.9
204	−74.5	−71.4	37.91
203	−68.4	−57.9	50.78
HecE	−71.1	−60.9	72.88

All the known CDKL5 5′UTR variants were analyzed (see sequences in Supplementary material 1). ΔGMFE (MFE, minimum free energy), ΔGCE (CE, ΔGMFE centroid), and ED (ensemble diversity) indices are reported. The sequences showing a difference of 5 points between the ΔGMFE and the ΔGCE index and an ED value not exceeding the threshold of 50 were considered to have a reliable prediction. The 205 and 202 first exon sequences have the best predicted structures.

### 3.3. eIF4B silencing reduces the expression of CDKL5

**Figure 6:**
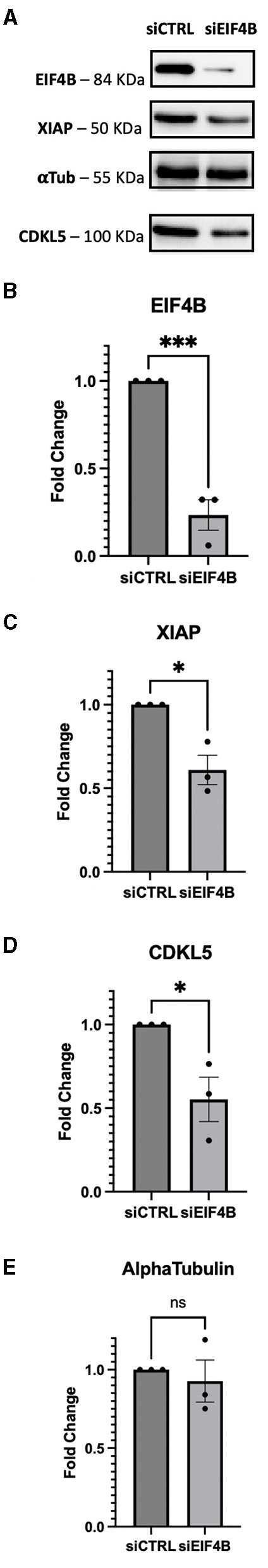
SHSY-5Y cells were transfected with siRNA against eIF4B and incubated for 72 h. **(A)** XIAP was used as positive control for the effect of eIF4B gene silencing; ponceau was used as internal reference for normalization of loading and αtubulin were considered as negative controls according to Shahbazian et al. (2010). **(B)** Upon silencing, the protein level of eIF4B decreased by 80% (*p*-value = 0.0009) **(C)** XIAP expression levels were reduced by 50%, as expected (*p*-value = 0.0112). **(D)** CDKL5 protein levels showed a decrease of 50% (*p*-value = 0.0282). **(E)** On the contrary, the positive control αtubulin does not show a significant decrease (*p*-value = 0.6165), maintaining its steady levels even when protein eIF4B is silenced. siCTRL, sample obtained from cells transfected with a random siRNA, used as negative control; sieIF4B, sample obtained from cells transfected with sieIF4B. The decreases were statistically significant, as determined by unpaired Student-t test analysis [*p*-value < 0.05 (^*^), *P*-value < 0.01 (^**^), *p*-value < 0.001 (^***^)].

Because of the highly structured 5′UTRs of CDKL5, we hypothesized a translational regulation by eIF4B modulation of the DEAD-box eIF4A helicase (Dmitriev et al., 2003; Shahbazian et al., 2010; Parsyan et al., 2011; Sen et al., 2016). Since eIF4A is an obligatory component of the translational machinery (see, for instance, Sénéchal et al., 2021), we could not consider a gene-silencing approach related to this protein. Therefore, we focused on eIF4B, and we assessed if the downregulation of this translation initiation factor could inhibit CDKL5 expression. To test this hypothesis, we examined CDKL5 protein levels in lysates obtained from control and eIF4B-silenced SHSY5Y cells. We included in the analysis another X-linked apoptosis regulator, XIAP (*NM*_001167.4: ΔGMFE 5′UTR = −133.60 kcal/mol, GC% = 65.2), as a positive control (Holcik et al., 2000). In fact, XIAP was reported to be translationally underexpressed in Hela cells when eIF4B was silenced (Shahbazian et al., 2010). The result shows that transfection of eIF4B siRNA (Figures 6A, B) halved the expression of CDKL5 as well as of XIAP (Figures 6A, C, D). On the contrary, the levels of αtubulin, a protein with unstructured 5′UTR (*NM*_001101.5: ΔGMFE 5′UTR = 13 kcal/mol, GC% = 76.2), used as negative control (Shahbazian et al., 2010), remained unchanged over treatment (Figures 6A, E).

### 3.4. CDKL5 5′UTR does not inhibit the translation efficiency of a downstream ORF

**Figure 7:**
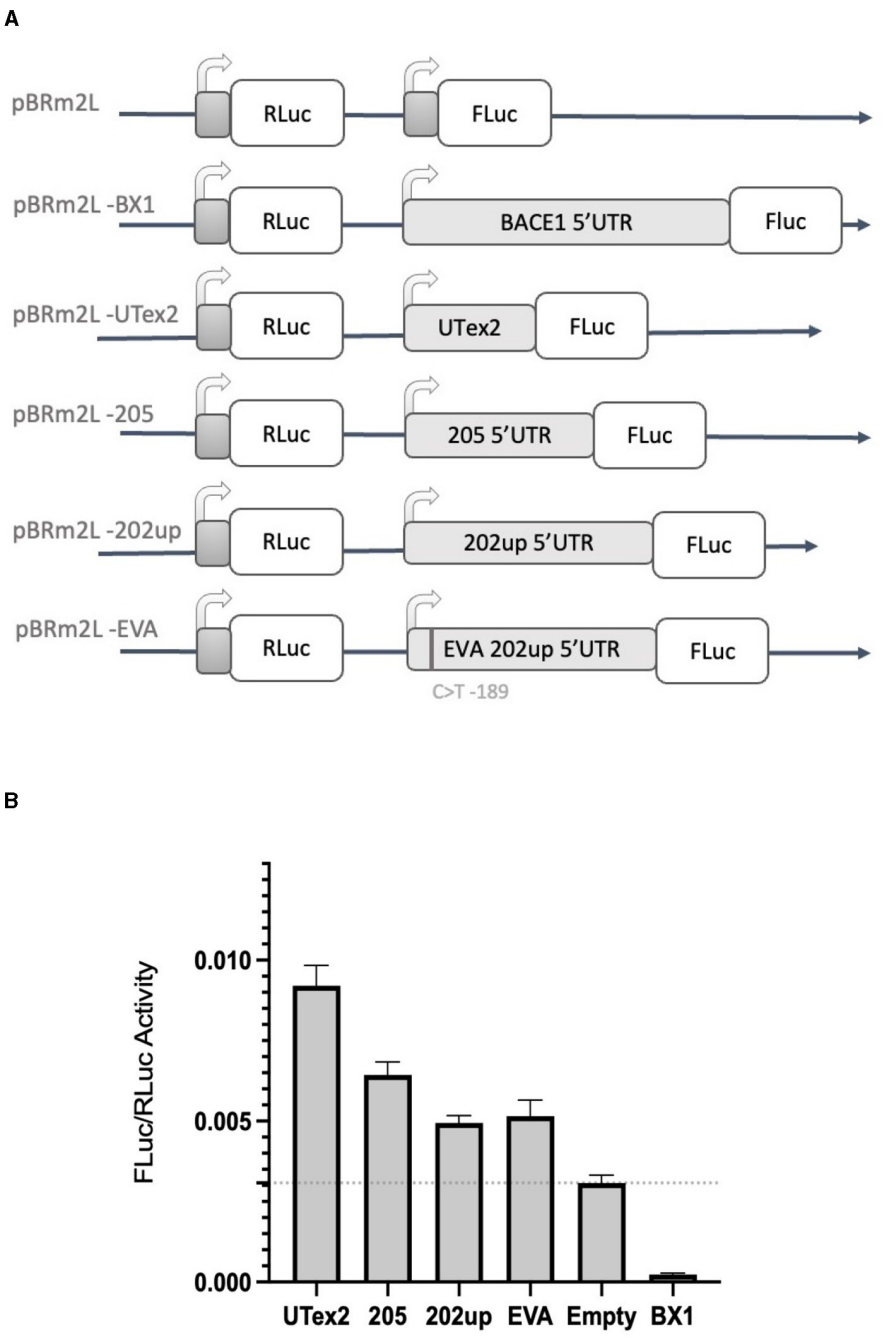
Dual luciferase reporter assay with the two-promoter vector. **(A)** Diagram of the two-promoter vectors in which the coding sequences of two luciferase genes are under the control of two distinct T7 promoters: pBRm2L is the control empty vector, while the other plasmids contains respectively. pBRm2L-UTex2 the 162 nucleotides UTex2 sequence; pBRm2L-205, the complete reference transcript leader of the CDKL5 variant 205; pBRm2L-202up, the complete transcript leader 202 with the addition of the 10 upstream nucleotides resulted from the RT-PCR analysis; pBRm2L-EVA, the same transcript leader sequence cloned in the vector pBRm2L-202up, but it carries the SNP rs786204994 (Evans et al., 2005); pBRmL2-BX1, the BACE1 5′UTR (De Pietri Tonelli et al., 2004). **(B)** The FLuc/RLuc ratios obtained from transfection with the vectors reported in **(A)**. All the CDKL5 5′UTRs showed an increase in the translational efficiency of FLuc. pBRmL2-EVA did not show any difference when compared to its control pBRmL2-202up. BACE1 5′UTR was used as positive control. pBRm2L was used to set the baseline translation. The experiment was repeated three times independently, analyzing two biological independent samples for each vector in each experiment (*n* = 6). Error bars represents SEM index.

With the aim of assessing the capability of the selected 5′UTR variants (205 and 202up) to modulate the translation of a downstream protein, we performed a dual-luciferase reporter assay. In the first experiment, we employed the “bi-monocistronic” two promoter vector pBRm2L (De Pietri Tonelli et al., 2004), that contains two independent transcriptional units under the control of distinct T7 RNA polymerase promoters (Figure 7A). Infecting the cellular system with the MVA virus carrying the T7 RNA polymerase gene, allowed to obtain the rapid, cytosolic transcription of the two cistrons, eliminating in this manner some of the technical limitations, such as the presence of cryptic promoters or splicing events, traditionally linked to standard DNA transfection when translation has to be assessed (Yang and Wang, 2019). The first cistron of the vector encodes the Renilla Luciferase (RLuc) protein downstream a short optimized 5′UTR, while the second cassette encodes the Firefly Luciferase (FLuc) placed downstream the 5′UTRs of interest. Based on the premise of the bioinformatic analysis, we analyzed the 205 (250 nts) and 202up (285 nts) CDKL5 transcript leaders (Figure 7A). Translation efficiency was quantified as the ratio of FLuc and RLuc signals. The sequence under analysis were compared with three controls: (i) the empty pBRm2L vector in which the expression of FLuc is under the control of short, optimized 5′UTR; (ii) the pBRm2L-B1x vector containing the transcript leader of BACE1 (GenBank accession number BG833894), a sequence known to reduce the translation efficiency of the downstream ORF (De Pietri Tonelli et al., 2004); and (iii) a truncated version of the CDKL5 5′UTR composed uniquely by the untranslated region of exon two (162 nts, UTex2), in order to evaluate, by difference, the effect of the first exons on overall translation of CDKL5 (Figure 7A). This reporter gene approach revealed that all the CDKL5 5′UTRs analyzed (205, 202up, UTex2, respectively, cloned in pBRm2L-205, pBRm2L-202up, and pBRm2L-UTex2) did not inhibit the Fluc expression, as the BACE1 5′UTR did, but rather caused an increase in translation efficiency (UTex2: +195%; 205: +119%; 202up: +57% compared to the baseline set by pBRm2L; Figure 7B, statistical analysis in Supplementary Table S3). This result suggests the presence of a cis-acting regulatory motif working as an enhancer of the translation efficiency in the CDKL5 5′UTR, spanning from at least the sequence Ex1 205 to the UTex2. At the same time, we tested the effect of the SNP rs786204994 described by Evans et al. (2005), with the aim to assess a possible inhibitory effect (by the use of the construct pBRm2L-EVA). However, choosing the condition 202up as control, we did not observe any significant difference (Figure 7B).

### 3.5. The CDKL5 5′UTR can support non-canonical translational initiation

**Figure 8:**
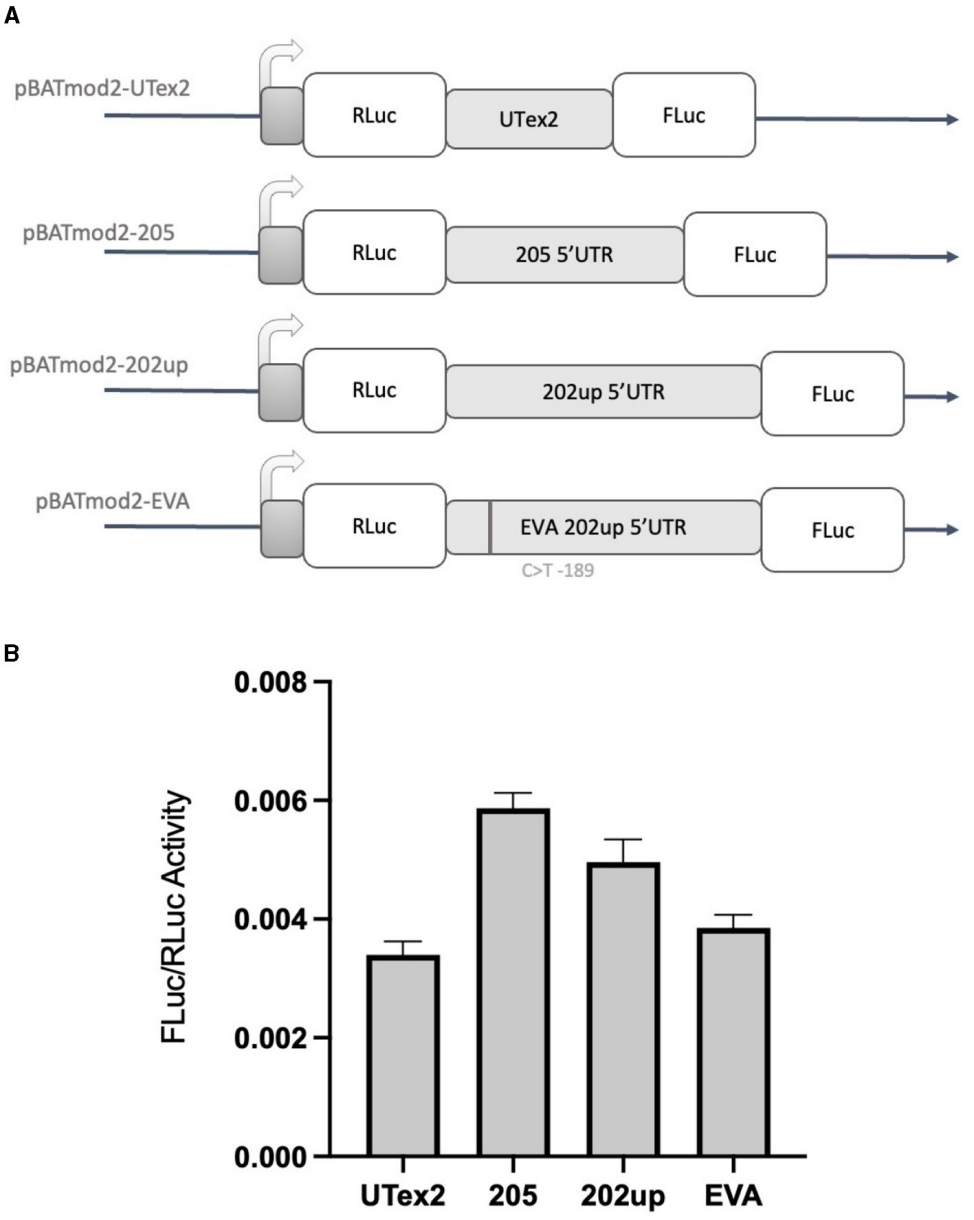
Dual-luciferase reporter assay with a dicistronic vector. **(A)** Schematic representation of the dicistronic vectors with the corresponding inserts: pBatmod2-205, pBatmod2-202up, pBatmod2-UTex2, and pBatmod2-EVA. **(B)** FLuc/RLuc ratios after transfection of the vectors described in **(A)** showing that translation of the second cistron is supported in all the vectors analyzed, with the different degrees of non-canonical translation initiation. The experiment was repeated three times independently, analyzing two biological independent samples for each vector in each experiment (*n* = 6). Error bars represents SEM index.

Based on the results obtained with the “bi-monocistronic” approach, we considered the presence of a cis-acting enhancing motif in the CDKL5 transcript leader that could have IRES features, allowing CDKL5 transcripts to undergo not only cap-dependent but also cap-independent translation. This dual mode of manage translation is not unusual for neuronal transcripts with important function in development (Audigier et al., 2008; Choi et al., 2019). Therefore, to verify the assumption that also CDKL5 could act in a similar manner, we performed the dual-luciferase reporter assay using the dicistronic pBatmod2RL vector. This construct allows to investigate the presence of a cap-independent translation that involves the presence of an IRES (De Pietri Tonelli et al., 2003). In this vector, the two luciferases coding regions are under the control of a unique T7 promoter that allows the cap-dependent initiation of the first ORF (RLuc) but not of the second one (FLuc), leaving the translation of the latter under the control of non-canonical, cap-independent translational initiation. In this experiment, we analyzed the 205, 202up, UTex2 transcript leaders, as well as the one carrying the rs786204994 SNP (Figure 8A). The FLuc/RLuc ratio values obtained confirmed the presence of an enhancing cis-acting regulatory element involving in cap-independent initiation (Figure 8B). In this case, the values obtained for the 205 and 202up transcript leaders were higher than the one returned from the UTex2 (205: +52.5% and 202up: +28.8% compared to UTex2), suggesting a contribution of the first exon sequence in the IRES function, regardless the presence of the 5′ extension of the 202up variant (Figure 8B, statistical analysis in Supplementary Table S4). Interestingly, rs786204994 SNP described a patient in the 5′UTR of CDKL5 (Evans et al., 2005) produced a decrease of about 25% on the FLuc/RLuc ratio when compared to its control *pBatmod*2_202*up* (Figure 8B). This finding suggests that C189 position is involved in the enhancing regulatory motif and that its transition to T might impair the non-canonical translational initiation efficiency, suggesting a possible mechanism in CDD pathogenesis. In any case, the disturbing effect of the C>T transition at position −189 agrees with its predicted effect on Δ*G*, which becomes less negative compared to the Δ*G* variations of all the possible C>T transitions in the CDKL5 5′UTR sequence (Supplementary Figure S1).

### 3.6. Quantification of the CDKL5 TSSs provides the percentage of usage of each alternative first exons

**Figure 9:**
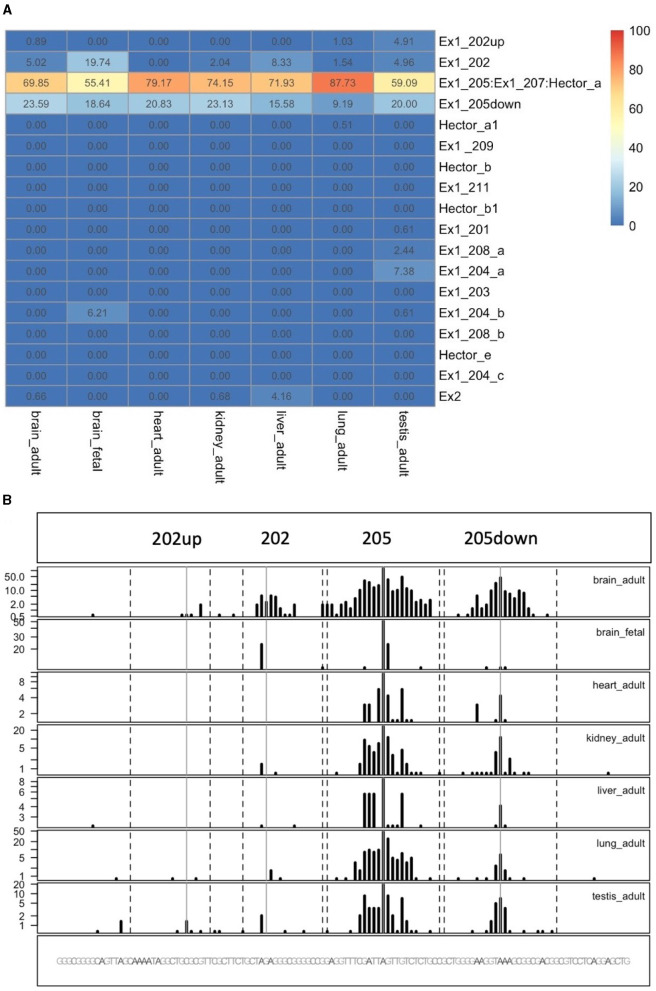
Quantification of CDKL5 TSSs in human tissues. **(A)** Analysis of CDKL5 TSS frequency in PHANTOM 5 CAGE libraries expressed as percentage of use heatmaps. Genome coverage is reported in **(B)**. The most abundant CDKL5 TSS belongs to Ex1 205 (ChrX: 18425608), Ex1 205down (ChrX: 18425633), Ex1 202 (ChrX: 18425583), and 202up (ChrX:184255573). Other TSSs are weakly expressed and consequently the other alternative first exons resulted to be poorly represented in the composition of the transcript leader. The testis is the tissue in which the CDKL5 transcription starts from more alternative TSSs, even if their percentage of usage remains low. Notably, transcription of CDKL5 in the adult brain, kidney and liver can also start from exon 2 (percentage of usage = brain: 0.66%; kidney: 0.68%, liver: 4.16%).

**Figure 10:**
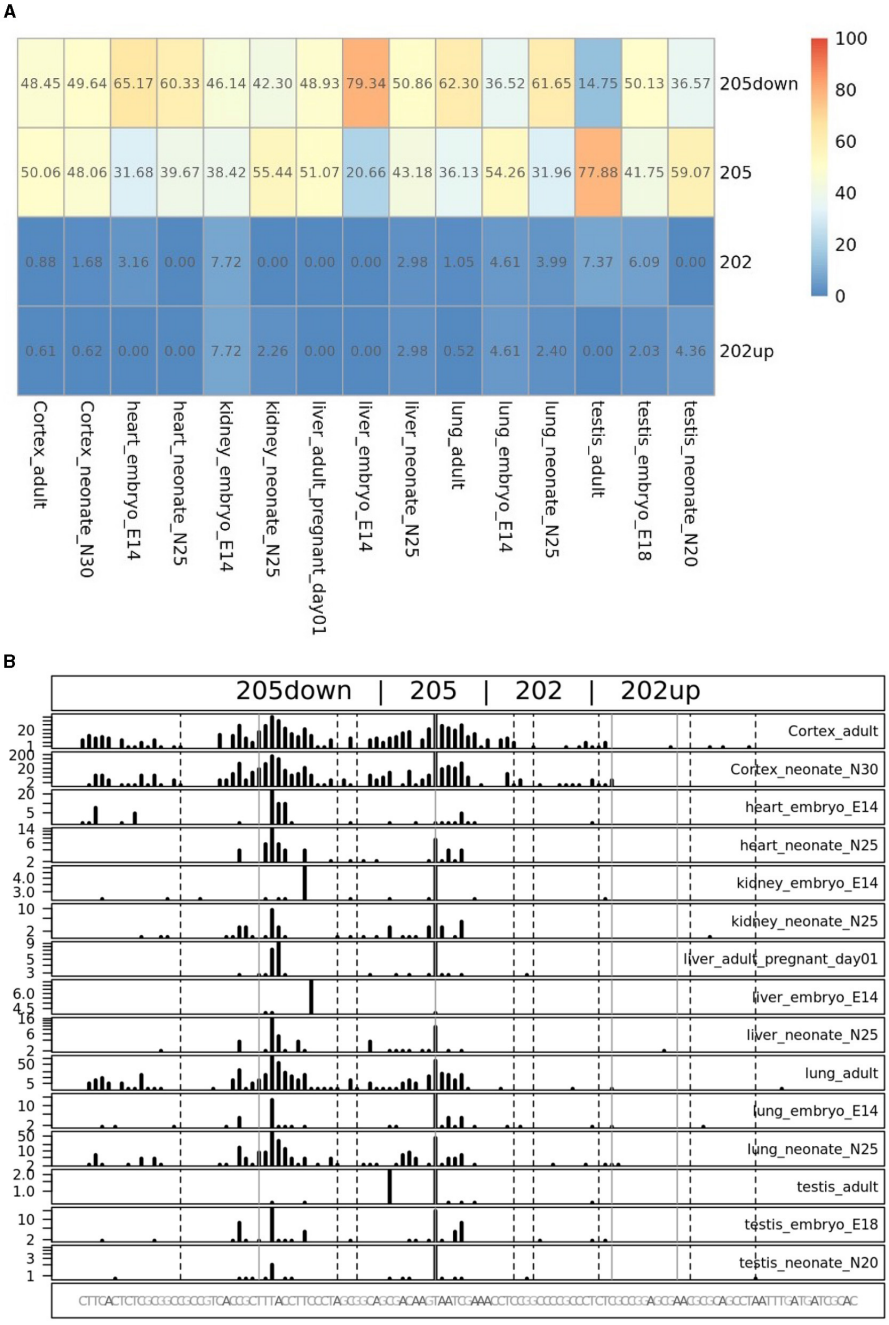
TSS peak quantification in murine FANTOM 5 libraries. Murine FANTOM 5 libraries obtained from various tissues (cortex, heart, kidney, liver, lung, and testis) were analyzed at different stage of development (adult, neonate and embryo, when available). **(A)** Levels are expressed as percentage of use heatmaps. Genome coverage is reported in **(B)**. The analysis confirmed the presence of all the four TSSs described in human transcriptomes. However, two glaring differences are notable: (i) the greater involvement of the 205down TSS in CDKL5 transcription, drawing the percentage of usage of the 205 TSS and even exceeding it in some tissues (e.g., in liver in the E14 embryo); (ii) the lower expression of the 202 and 202up TSSs, without any significant difference in term of percentage of use in different developmental stage.

To quantify the abundance of the variants carrying the 205 and 202up CDKL5 5′UTRs, we analyzed the percentage of the CDKL5Transcription Start Sites (TSSs) in various adult human tissues by consulting the FANTOM 5 libraries (Noguchi et al., 2017). The evaluation of the cap analysis of gene expression (CAGE) libraries can determine the precise positions of the CDKL5 TSSs, solving the problem of the ambiguous 5′limits described previously. For our analysis, we selected libraries from the following adult organs: brain, heart, kidney, lung, spleen, liver, testis, and fetal brain. This last library was added to assess the presence of an alternative first exon usage in different developmental stages according to the difference in the CDKL5 expression during brain development (Rusconi et al., 2008). The quantification was conducted using the CAGEr package from bioconductor (Haberle et al., 2015). The results show that most of the alternative CDKL5 first exons reported in Ensembl and in the study of Hector et al. (2016) are almost absent in the analyzed transcriptomes since their TSS were not detected at all. On the contrary, the TSS of Ex1 205 (NM001323289/NM003159.3) resulted to be the preferential CDKL5 TSS in each tissue analyzed, ranging from a minimum of percentage of usage of 55.41% in the fetal brain and a maximum of 87.73% in the lungs (Figure 9A). The other reference TSS, belonging to Ex1 204a (NM 001037343) resulted to be expressed uniquely in the testis (the human tissues with more used TSSs), with a percentage of usage of 7.38%. The second most abundant TSS was represented unexpectedly by an unreported TSS lying 26 nucleotides downstream the TSS of 205, that we called 205down (Figure 9B). Its existence is confirmed by the CAGEr peak quantification analysis conducted in various murine tissues, where it has a more weight in determining CDKL5 transcription, with a percentage of usage greater of TSS of 205 in some tissues (Figure 10A). In human tissues, the frequency of transcription of the Ex1 205down averages 20% (max. adult kidney: 23.12%; min. adult kidney: 9.19%). The resulting first exon sequence differs from the longer Ex1 205 from the exclusion of the *pG*4_2_ motif, suggesting a possible functional difference in the structure of the transcript leader or in the definition of the promoter. The other TSS that we identified, the TSS of 202up, previously assumed after the RT-PCR (Figure 9), was less expressed (max. adult testis 4.91%), contrary to the 10 nucleotides downstream the TSS of 202 that is the third most transcription starting point of CDKL5, with a maximum of 19.74% in fetal brain, while it ranges between 0 and 8.33% in adult tissues (Figure 9A). The comparison of the percentage of use level of the TSS of 202 in the adult brain and fetal brain (adult: 5.02; fetal: 19.74%) at the expense of the TSS of 205 (adult: 69.85%; fetal: 55.41%) point toward a plausible differential transcriptional preference never observed in any other adult tissues and hinting a possible role in brain development (Figures 9A, B). This information might turn out to be interesting since we did not observe a similar trend in the murine cortical libraries (taken into consideration in the absence of total brain libraries), where the percentage of use of the TSS of 202 remains stable in a tight range in different developmental stages (adult: 0.99%; neonate: 1.21%; embryo: 0.53%; Figures 10A, B). Our analysis defined the presence of a unique TSS cluster for the gene CDKL5, composing by four start sites: 202up, 202, 205, and 205down (Figure 9B). The result obtained highlights that while the TSS cluster is conserved between the human and the mouse, the percentage of usage of the single TSSs highly differs. Despite the high conservation of the transcript leaders of CDKL5, our finding points toward the possibility to have an alternative use of the various TSS within an evolutionary context, favoring, in humans, longer 5′UTRs, enriched in cis-acting elements.

## 4. Discussion

### 4.1. 5′UTR variants of CDKL5

The growing relevance of CDKL5 as a player in neuronal functions highlights the need of gaining knowledge about the regulation of its expression. In this context, translational regulation seems to have a crucial role based on hints available in the literature (La Montanara et al., 2015) as well as on the complexity of the transcript leader of CDKL5. This necessity arises also from the need to improve the diagnostic accuracy of CDD since some of the untranslated exonic regions of CDKL5 were routinely excluded from the diagnostic screenings because they are considered unfunctional (Hector et al., 2017a,b) although SNPs with possible pathological consequences have been described (Nemos et al., 2009; Mei et al., 2010). A Part of the difficulties in investigating CDKL5 5′UTR is due to the inaccuracy in the definition of the variant sequences and, consequently, to the lack of a common nomenclature. For this reason, we first propose an unambiguous nomenclature based on the Ensembl transcript IDs and the experimental study of Hector et al. (2016). From the possible 5′UTRs emerged that the numerous variants of the 5′UTR maintain an established sequence organization, sharing a sequence proximal to the start codon (UTex2) in the second exon but varying in the first exon(s). This further highlights the presence of various TSSs, as previously hypothesized (Hector et al., 2016). However, the difference of few nucleotides at the 5′ ends of some alternative first exon sequences suggests the presence of errors in the reported variants possibly due to the technical limitation in the determination of the TSSs of the gene (Hector et al., 2016; Adiconis et al., 2018). Our analysis confirmed this possibility, showing that the majority of the CDKL5 first exon variants are not conserved or not reported in the transcriptomes of non-primate species, and their TSSs are absent in FANTOM5 libraries. By CAGEr (Haberle et al., 2015), we validated a TSS cluster around the reference TSS 205 (ChrX: 18425608), composed by other three TSSs closely spaced on the same region, named 202up (ChrX: 18425573), 202 (ChrX: 18425583) and 205down (ChrX: 18425633). Although CDKL5 TSSs identification has been previously attempted (Vitezic et al., 2014; Hector et al., 2016), our CAGE analysis provided new elements to the topic, such as the differential percentage of usage of the variants in the context of human development (adult vs. fetal). Moreover, this approach hints to a multifaceted and dynamic interplay between CDKL5 transcriptional and translational mechanisms with a possible inclusion of part of the transcript leader in the composition of the promoter and vice versa. This agrees with the complex temporal expression pattern of the 5′UTR of CDKL5 in various tissues.

### 4.2. EIF4B is involved in the translational regulation of CDKL5

From a functional perspective, our bioinformatical evaluation predicted a richness of structural cis-acting elements in this exonic region of interest, with a stable global structural folding of the 5′UTR. This feature points to the involvement of the DEAD-box helicase eIF4A and its modulation by eIF4B (Parsyan et al., 2011). Since a strong and prolonged gene silencing of eIF4A, an essential protein of the translational machinery (Sénéchal et al., 2021), would have affected the cell physiology in a complex and unpredictable manner, we decided to focus on the downregulation of eIF4B. Indeed, our hypothesis of the engagement of these motifs in the 5′UTR-mediated translational control of CDKL5 was confirmed by the involvement of this translational initiation factor in setting the CDKL5 protein level. This correlation is particularly relevant in the neuronal context, given the established role of eIF4B in the dynamic modulation of the translation initiation rates (Harms et al., 2014; Sen et al., 2016), and its proposed role in local translation (Bettegazzi et al., 2017) at the level of synapses.

### 4.3. Loss-of-function C>T -189 mutation in CDKL5 5'UTR suggests the presence of a non-canonical translational regulation mechanism

eIF4B is involved in both cap-dependent and cap-independent translational initiation (López de Quinto et al., 2001). While we suggest the presence of a translation control mediated by the 205 and 202up 5′UTRs that is consistent with the eIF4B function in cap-dependent translation, we also found that the CDKL5 5′UTR could support cap-independent translational initiation that enhances the expression of the downstream ORF. More specifically, we propose the presence of an IRES in the CDKL5 transcript leader that, according to the prediction of structural elements, the analysis of the evolutionary conservation, and the results obtained with the UTex2 5′UTR (i.e., a truncated version of 205 and 202up) could span from UTex2 to the proximal part of the first exon. The possible presence of this non-canonical mechanism in CDKL5 translation makes this kinase to fall in roughly 17% of human transcripts able to switch from cap-dependent to the IRES-mediated initiation according to the cellular needs and occurring frequently in brain and neuronal development (Audigier et al., 2008; Choi et al., 2018; Fan et al., 2022). Interestingly, and in agreement with this hypothesis, we used our experimental approach to collect the first evidence of the effect of the transition C>T-189 identified in a CDD patient (Evans et al., 2005). To be associated with the CDD condition, this SNP would be expected to decrease the expression of the downstream ORF. Indeed, we were able to observe this effect when cap-independent translation initiation was assessed, hinting to the inclusion of C-189 in the proposed CDKL5 IRES. IRESes are usually structural elements that can be formed by either Watson and Crick base pairing or Hoogsteens G quadruplex assembly (Morris et al., 2010; Hoque et al., 2022). Our approach predicted both structures in the analyzed 5′UTR of CDKL5. However, the evidence collected by the effect of the C>T-189 SNP on translation and the stem-loop structural context in which this SNP lies points toward a Watson and Crick base pairing in the composition of the IRES. In fact, a Hoogsteens-based in the structure *pG*4_2_ that would support the IRES structure would be unaffected or even reinforced by the C>T-189 SNP, while our experimental evidence with luciferase reporter genes is against this hypothesis.

### 4.4. Conclusions and perspectives

In our study, we characterized the features of the 5′UTR of CDKL5, demonstrating its potential role in the translational regulation of the downstream protein. Moreover, we provide evidence that a SNP in this region might play a pathological role. SNPs affecting translation efficiency are becoming a growing finding in genetic analysis, especially in neurodevelopmental disorders (see, for instance, Pan et al., 2021; Coursimault et al., 2022). Interestingly, the presence of active cis-acting regulatory elements in the transcript leader could be exploited to boost the expression of the target protein by using approaches such as antisense oligonucleotides (ASOs) (Liang et al., 2017). ASOs, highly selective, minimal invasive, and customizable tools, are already emerged as treatment for loss-of-function monogenetic disorder (Bennett, 2019). Recently FDA approved one ASO (nusinersen) for Spinal Muscular Atrophy (Li, 2020) and ASOs targeting the 5′UTR has shown to be a promising treatment for cystic fibrosis (Sasaki et al., 2019). Therefore, a better understanding CDKL5 5′UTR will improve not only the accuracy of the diagnostic health care but also pave the way to a new therapeutical strategy to treat CDD by altering the efficiency of translation.

## Data availability statement

The raw data supporting the conclusions of this article will be made available by the authors, without undue reservation.

## Ethics statement

The animal study was approved by IACUC of the San Raffaele Scientific Institute (Auth. n.1077, Ministry of Health). The study was conducted in accordance with the local legislation and institutional requirements.

## Author contributions

VR conceived the study, performed experiments and bioinformatic analysis, analyzed data, prepared figures, and wrote the manuscript. CF and VDC supervised and performed experiments. SdP supervised and performed bioinformatic analysis. NL discussed the experiments and participated in manuscript writing. DZ conceived and designed the study, supervised the experiments and the analyses, and wrote the manuscript. All authors contributed to the article and approved the submitted version.
