# Supplementary material for: Complex CDKL5 translational regulation and its potential role in CDKL5 deficiency disorder
Source: Front Cell Neurosci. 2023 Oct 30;17:1231493. doi: 10.3389/fncel.2023.1231493 (PMC10642286; doi:10.3389/fncel.2023.1231493)
Supplement: Supplementary file 1 [file Data_Sheet_1.PDF]

```
>Homo_sapiens
Hsap
GCTTCTGCTAGAGGGCGGGGCCGGAGGTTTCGATTAGTTGTCTCTGCCGCTGGGGAAGGTAAAGCGGC
GACGGCGTCCTCAGGAGCTGTGGGGTCCCCTGCTAGAAGTGGGGGACTCGGCGGGGGAGTCATTTAAT
ACTTCATGATTAGAACAAATATGTGAAAGTTCCCACCAACCAGTGAGAATTTCTTCCTTCAGACGGTT
TTGGATCTTACTGCACAGCTTTCTGAGAAGTTCTTTTGGTGCCATGTTTTGTGGCTTGCATCAAAAGA
GGAGTTTGTCTTCATGAAGATTCCTAACATTGGTAATGTGATGAATAAATTTGAGATCCTTGGGGTTG
TAGGTGAAG


>Mus_musculus
Mmus
GCTCCGGCGAGAGGGCGGGGCCGGAGGTTTCGATTACTTGTCGCTGCCGCTAGGGAAGGTAAAGCGGT
GACGGCGGCCGCGAGAGTGAAGGCTTCCCCGATAGAGGTGGGGGACTCGGCAGGGGACTCAGTTAATA
CTGCACAGTTGAACAAATGTATGAAGGTTCCTTGCCAACCATTGAGAATTTCTTCGTTGGGACATTAT
TTCCCTGCTTACTGAATAACTTGTTTAGACGTTTCTTTTTTGGTGCCATGTTCTGTGGCTCACATCAA
AAGAGGAGTTTGTCTTCATGAAGATTCCTAACATTGGTAATGTGATGAATAAATTTGAGATCCTTGGG
GTTGTCGGTGAAG


>Lynx_canadiensis
Lcan
GCCTCTGCGAGAGGGCGGGGCCGGAGGTTTCCATTAGTTGTCCCTGCCACTGGGAAAGGTAAAGCGGC
GACGGCGGCCTCAGGAGCTGTGGGGTCCCCGGCTGGAGGTGGGAGACTCGGCGGGGGAATCATTTAAT
ACTTCACATTACAACAAAATGTGAAAGTTCCTGCCAACCTGTGAGAATTTCTTCCTTCAACATTTTCT
ATTTACTGCACAACTTTCTCAGTTCTTTTTTGATGCCATGTTTTGTGGCTTGCATCAAAAGAGGAGTT
TGTCTTCATGAAGATTCCTAACATTGGTAATGTGATGAATAAATTTGAGATCCTTGGGGTTGTAGGTG
AAG


>Lagenorhynchus_obliquidens
Lobl
GCCTGAGAGAGAGGGCGGGGTCGAAGGTTTCGATTAGTTGTCGCCGCCGCTGGGGAAGGTAAAGCGGC
GACGGCGGCCTCGGGAGCTGTGGGACCCTGGCTGGAGGTGGGGATCTCGGCGGGGGAATCATTTAATA
CTTCACATTAGGACAAATATGTGAAAGTTCCTGCCAACCTGTGAGAATTTCTTCCTTTGGACATTTTT
TGTTCTACTTACTGTACAACTTTCTTAGAAGTTCTTTTTTGGTGCCATGTTCTGTGGCTTGCATCAAA
AGAGGAGTTTGTCTTCATGAAGATTCCTAACATTGGTAATGTGATGAATAAATTTGAGATCCTTGGGG
TTGTAGGTGAAG


>Ictidomys_tridecemlineatus
Itri
TCTTCTGCGAGAGGGCGGGGCCGGAGGTTTCGATTAGTTGTCCCTGCCGCTGGGGAAGGTAAAGCGTC
GACGGCGGCTTCTGGAGCAGTGGGGTCCCCGGCTGGAGGTGGGGACTCAGCGGGGAACTCATTTAATG
CTTCATTATTACAACAAATATGTGAAAATTCCTGTCAACTGTTGAGAATTTTTTCCTTTGGATATTGT
TTCCTCCATTTACTGCATAAATTTCTTAGAAAAAAAATTTTTTTTGGTGCCATGTTCTGTGGCTTACA
TCAAAAGAGGAGTTTGTCTTCATGAAGATTCCTAACATTGGTAATGTGATGAATAAATTTGAGATCCT
TGGGGTTGTAGGTGAAG


>Monodelphis_domestica
Mdom
GCCCGGTCGGGAGGGCGGGGCCGGAGGGTTCGATTAGTTGCCACTGCCGCTGGGGAAGAGAAAACAGC
GACGGCTGCGGCGGCTGTAGCGGTGGGAGTCCCCGCTTAAAGCCGGGACTGAGTGGGGAGGAATCATT
```

TTATGCTTCAAAATTAGGACAAATATGTGAGAATTTCCATCAGCCAGCGTGGATTTCTTTCGTCAAGC
TCTTCAAATACAACTCACTATATAACTTTCTCAGATAACCTTCTCTGTGCCATGTTCTGTGGCTTGCA
TCAAAAGAGGAATTTGTCTTCATGAAGATTCCTAACATTGGTAATGTGATGAATAAATTTGAGATCCT
TGGGGTTGTAGGTGAAG

>Sarcophilus_harrisii
Shar
GCCCGGTCGGGAGGGCGGGGTCGGAGGATTCGATTAGTTGCCACTGCCGCTGGGGTAGAGAAAACAGC
GACGGCTGCGGCGGCTGTAGCGGTGGGAGTCCCCGCTTAGAGCTGGGATAGAGTGGGGGGGAATCATT
TTATACTTCAGAATTAGGACAAGTATGTGAGAATTCCCATCAGCCAGCGTGGATTTCTTTCTTCAAGT
TCTTCCAATACAGCCAGCTACATAACTTTCTCAAATAACCCTTCTTTGTGCCATGTTCTGTGGCTTGC
ATCAAAAGAGGAATTTGTCTTCATGAAGATTCCTAACATTGGTAATGTGATGAATAAATTTGAGATCC
TTGGGGTTGTAGGTGAAG

>Monodon_monoceros
Mmon
GCCTGAGAGAGAGGGCGGGGTCGAAGGTTTCGATTAGTTGTCGCCGCCGCTGGGGAAGGTAAAGCGGC
GACGGAGGCCTCGGGAGCTGTGGGACCCTGGCTGGAGGTGGGGATCTCGGCGGGGGAATCATTTAATA
CTTCACATTAGGACAAATATGTGAAAGTTCCTGCCAACCTGTGAGAATTTCTTCCTTTGGACATTTTT
TGTTCTACTTACTGTACAACTTTCTTAGAAGTTCTTTTTTGATGCCATGTTCTGTGGCTTGCATCAAA
AGAGGAGTTTGTCTTCATGAAGATTCCTAACATTGGTAATGTGATGAATAAATTTGAGATCCTTGGGG
TTGTAGGTGAAG

>Acinonyx_jubatus
Ajub
GCCTCTGCGAGAGGGCGGGGCCGGAGGTTTCCATTAGTTGTCCCTGCCACTGGGAAAGGTAAAGCGGC
GACGGCGGCCTCAGGAGCAGTGGGCTCCCCGGCTGGAGGTGGGAGACTCGGCGGGGGAATCATTTAAT
ACTTCACATTACAACAAAATGTGAAAGTTCCTGCCAACCTGTGAGAATTTCTTCCTTCAACATTTTCT
ATTTACTGCACAACTTTCTCAGTTCTTTTTTGATGCCATGTTTTGTGGCTTGCATCAAAAGAGGAGTT
TGTCTTCATGAAGATTCCTAACATTGGTAATGTGATGAATAAATTTGAGATCCTTGGGGTTGTAGGTG
AAG

>Camelus_dromedarius
Cdro
GCCTGTGCGAGAGGGCGGGGTCGAAGGTTTCGATTAGTTGTCGCCGCCGCTGGGGAAGGTAAAGCGGC
GACGGCGGCCTCAGGAGCTGTGGGGTCCATGGCTGGAGGTGGGGGACTCGGCGGGAGAATCATTTAAT
ACTTCATATCAGGACAAATATGTGAAAGTTCCTGCCAACCTGTGAGAATTTCTTCCTTTGGACATTTT
TCCTTCTACTTACTGTACAACTTGCTAAGAAGTTCTTTTCTGATGCCATGTTCTGTGGCTTGAATCAA
AAGAGGAGTTTGTCTTCATGAAGATTCCTAACATTGGTAATGTGATGAATAAATTTGAGATCCTTGGG
GTTGTAGGTGAAG

>Equus_caballus
Ecab
GCCTCTGCGAGAGGGCGGGGTCGGAGGTTTCGATTAGTTGCTGCCGCTGGGGAAGGTAAAGCGGCGAC
GGCGGCCTCAGGAGCTGAGGGGTCCCCGGCTGGAGGCGGGGGACTCGGCGGGGGAATCAGTTAATGCT
TCGCAGTATAACAAATATGTGAAAGTTCCACCAAACTGTGAGAATTTCTTCGGACATTTTTTCGTTCT
ATTTACTGCACAACTTCATCGAAGTTCTTTTTTTAATGCCATGTTCTGTGGCTTGCATCAAAAGAGGA
GTTTGTCTTCATGAAGATTCCTAACATTGGTAATGTGATGAATAAATTTGAGATCCTTGGGGTTGTAG
GTGAAG

>Canis_lupus_dingo
Clupd
GCCTCTGCGAGAGGGCGGGGCCGGAGGTTTCCATTAGTTGTCCCTGCCACTGGGAAAGGTAAAGCGGC
GACGGCGGCCTCAGGAGCTTGGGGTCCCCGGCTGGAGGTGGGGGACTCAGCGGGGGAATCATTTCATA
CTTCACATTTGAATAAATATGTGAAAATCCCTGTCAACCTGTGAGAATTTCTTCTTTCGACATTTTTG
TTCTACTTCACAACTTTCTCAGAAGTTCTTTCTTGATGCCATGTTCTGTGGCTTGCATCAAAAGAGGA
GTTTGTCTTATGAAGATTCCTAACATTGGTAATGTGATGAATAAATTTGAGATCCTTGGGGTTGTAGG
TGAAG

>Vulpes_vulpes
Vvul
GCCTCTGCGAGAGGGCGGGGCCGGAGGTTTCCATTAGTTGTCCCTGCCACTGGGAAAGGTAAAGCGGC
GACGGCGGCCTCAGGAGCTGTGGGGTCCCCGGCTGGAGGTGGGGGACTCAGCGGGGGAATCATTTCAT
ACTTCACATTTGAATAAATATGTGAAAATTCCTGTCAACCTGTGAGAATTTCTTCTGTCGACATTTTT
GTTCTACTTCACAACTTTCTCAGAAGTTCTTTCTTGATGCCATGTTCTGTGGCTTGCATCAAAAGAGG
AGTTTGTCTTCATGAAGATTCCTAACATTGGTAATGTGATGAATAAATTTGAGATCCTTGGGGTTGTA
GGTGAAG

>Nannospalax_galili
Ngal
GCTCCGGCCAGAGGGCGGGGTCGGAGCTTTCGATTAGTTGTCGCTGCCTTTGGGGAAGGTAAAGCAGT
GACGGTGGTCGCCGGAGCAGAGGCTTCCCGGTCTTGGAGGTGGGAGACGCGGTGGGGGACTCATTCAC
TACTTCACTGTTAGAACAGATGCGAGAAAGTTCCTGCCACACATTGAGGATTTCTTCCTTTGCACGTA
TTTTCCCCCTGCTTACTGCATAACTTTCTTGGAATTTCCTTTTTTGGTGCCATGTTCTGTGGCTTACA
TCAAAAGAGGAGTTTGTCTTCATGAAGATTCCTAACATTGGTAATGTGATGAATAAATTTGAGATCCT
TGGGGTTGTAGGTGAAG

>Globicephala_melas
Gmel
GCCTGAGAGAGAGGGCGGGGTCGAAGGTTTCGATTAGTTGTCGCCGCCGCTGGGGAAGGTAAAGCGGC
GACGGCGGCCTCGGGAGCTGTGGAACCCTGCCTGGAGGTGGGGATCTCGGCGGGGGAATCATTTAATA
CTTCACATTAGGACAAATATGTGAAAGTTCCTGCCAACCTGTGAGAATTTCTTCCTTTGGACATTTTT
TGTTCTACTTACTGTACAACTTTCTTAGAAGTTCTTTTTTGGTGCCATGTTCTGTGGCTTGCATCAAA
AGAGGAGTTTGTCTTCATGAAGATTCCTAACATTGGTAATGTGATGAATAAATTTGAGATCCTTGGGG
TTGTAGGTGAAG

>Tursiops_truncatus
Ttru
GCCTGAGAGAGAGGGCGGGGTCGAAGGTTTCGATTAGTTGTCGCCGCCGCTGGGGAAGGTAAAGCGGC
GACGGCGGCCTCGGGAGCTGTGGAACCCTGCCTGGAGGTGGGGATCTCGGCGGGGGAATCATTTAATA
CTTCACATTAGGACAAATATGTGAAAGTTCCTGCCAACCTGTGAGAATTTCTTCCTTTGGACATTTTT
TGTTCTACTTACTGTACAACTTTCTTAGAAGTTCTTTTTTGGTGCCATGTTCTGTGGCTTGCATCAAA
AGAGGAGTTTGTCTTCATGAAGATTCCTAACATTGGTAATGTGATGAATAAATTTGAGATCCTTGGGG
TTGTAGGTGAAG

>Ovis_aries
Oari

GCCTGAGCGAGAGGGCGGGGTCGAGGGTTTCGATTAGTTGTCGCTGCCGCTGGGGAAGGTAAAGCGGT
GACGGCGGCCTCAGGAGCTGTGGGGTCCCCGGCTGGAGGTGGGGAACTCGGCGGGGAAATCATTTAAT
AATTCACATTAGGACAAATATGTGAAAGTTCCTGCCAACCTGTGAATATTTCTTCCTTCGGACATTTT
TTGTTCTACTTACTGTACAACTTTCTTAGAAGTTCTTCTTTGATGCCATGTTCTGTGGCTTGCATCTA
AAGAGGAGTTTGTCTTCATGAAGATTCCTAACATTGGTAATGTGATGAATAAATTTGAGATCCTTGGG
GTTGTAGGTGAAG

>Macaca_mulatta
Mmul
GCTTCTGCTAGAGGGCGGGGCCGGAGGTTTCGATTAGTTGTCTCTGCCGCTGGGGAAGGTAAAGCGGC
GACGGCGTCCTCAGGAGCTGTGGGGTCCCCTGCTAGAAGTGGGGGACTCGGCGGGGGAATTGTTTAAT
ACTTCATGATTGCAACAAGTATGTGAAAGTTCCCACCAGCCAGTGAGAATTTCTTCCTTCAGAAGGTT
TTTGGTCTTACCGCGCAGCTTTCTGAGTTCTTTTGGTGCCATGTTTTGTGGCTTGCATCAAAAGAGGA
GTTTGTCTTCATGAAGATTCCTAACATTGGTAATGTGATGAATAAATTTGAGATCCTTGGGGTTGTAG
GTGAAG

>Ailuropoda_melanoleuca
Amel
GCCTCTGCGAGAGGGCGGGGCCGGAGGTTTTCATTAGTTGTCCCTGCCACTGGGTAAGGTAAATCGGA
GACGGCGGCCTCAGGAGCTGTGGGGTCCCCGGCTGGAGGTGGGGGACTCGGCTAGGGAATCATTTAAT
ACTTCACATTAGAACAAATATGTGAAAGTCCCTGCCAGCCTGTGAGAATTTCTTCCTTTGACATTTTG
TTTCTACTTCACAACTGTCTCTGAAGTTCTTTCTTGATGCCATGTTCTGTGGCTTGCGTCAAAAGAGG
AGTTTGTCTTCATGAAGATTCCTAACATTGGTAATGTGATGAATAAATTTGAGATCCTTGGGGTTGTA
GGTGAAG

>Callithrix_jacchus
Ejub
GCCTCTGCGAGAGGGCGGGGCCGGAGGTTTCCATTAGTTGTCCCTGCCACTGGGAAAGGTAAATCGGC
GACGGCGGCCTCAGGAGCTGTGGGGTCCCCGGCTGGAGGTGGGGGACTCGGCTGGGGAATCATTTAAT
ACTTCACATTAGAACAAATATGTGAAAGTCCCTGCCAACCTGTGAGAATTTCTTCCTTCGACATTTTG
GTTCTACTTCACAACTTCCTTAGAAGTTCTTTCTTGATGCCATGTTCTGTGGCTTGCATCAAAAGAGG
AGTTTGTCTTCATGAAGATTCCTAACATTGGTAATGTGATGAATAAATTTGAGATCCTTGGGGTTGTA
GGTGAAG

>Eumetopias_jubatus
Cjac
GCTTCTGCTAGAGGGCGGGGCAGGAGGTTTCGGTTAGTTGTCTGCTGTTGGGGAAGGTAAAGCGGAGA
CGGCGTCCTCAGGAGCCGTGGGGTCCCCTGCTAGAAGTGGGGGACTCGGCAGGGAAATCATTTAATAC
TTGATGATTAGAACAAATATGTGAAAGCTCCCACCAACCAGTGAGAATTTCTTCCTTCAGAAGGGTTT
TTGTTCTTACTGCACAGCTTTTTTGGTGCCATGTTTTATGGCTTGCATCAAAAGAGGAGTTTGTCTTC
ATGAAGATTCCTAACATTGGTAATGTGATGAATAAATTTGAGATCCTTGGGGTTGTAGGTGAAG

>Mastomys_coucha
Mcou
GCTCCGGCGAGAGGGCGGGGCCGGAGGTTTCGGTTACTTGTCGCTGCCGCTAGGGAAGGTAAAGCGGT
GACGGCGGCCGCGAGAGTGGAGGCTTCCCCGATAAGGTGGGGGACTCGGCAGGGGATTCATTTGATAC
ATCACAGTTGAACAAATGTATGAAAGTTCCCTGCCAATCATTGAGAATTTCTTCATTGGGACATTTTT
TTCCTGCTTACTGAATAACTTGTTTAGAAGTTCCGTTTTTGGTGCCATGTTCTGTGGCTCGCATCAAA
AGAGGAGTTTGTCTTCATGAAGATTCCTAACATTGGTAATGTGATGAATAAATTTGAGATCCTTGGGG

TTGTCGGTGAAG

>Aotus_nancymaae
Anan
GCTTCTGCTAGAGGGCGGGGCCGGAGGTTTCGGTTAGTTGTCTCTGCCGTTGGGGAAGGTAAAGCGGA
GACGGCGTCCTCAGGAGCTGTGGGGTCCCCTGCTAGAAGTGGGGGACTCGGCAGGGAAATCATTTAAT
ACTTGATGATTAGAACAAATATATGAAAGCTCCCACCAACCAGTGAGAATTTCTTCCTTCAGAAGGGT
TTTTGTTCTTACCGCACAGCTTTCTGAGAAGTTCTTTTGGTGCCATGTTTTATGGCTTGCATCAAAAG
AGGAGTTTGTCTTCATGAAGATTCCTAACATTGGTAATGTGATGAATAAATTTGAGATCCTTGGGGTT
GTAGGTGAAG

>Leptonychotes_weddellii
Lwed
GCCTCTGCGAGAGGGCGGGGCCGGAGGTTTCCATTAGTTGTCCCTGCCACTGGGAAAGGTAAATCGGC
GACGGCGGCCTCAGGAGCTGTGGGGTCCCCGGCTGGAGGTGGGGGACTCGGCTGGGGAATCATTTAAT
ACTTCACATTAGAACAAATATGTGAAAGTCCCTGCCAACCTGTGAGAATTTCTTCCTTCGACATTTTG
GTTCTACTTCACAACTTTCTTAGAAGTTCTTTCTTGATGCCATGTTCTGTGGCTTGCATCAAAAGAGG
AGTTTGTCTTCATGAAGATTCCTAACATTGGTAATGTGATGAATAAATTTGAGATCCTTGGGGTTGTA
GGTGAAG

>Pteropus_vampyrus
Pvam
GCTTTTGCGAGAGGGCGGGGCCAGAGGTTTCGTTTAGTTGTCCCTGCAGCTGGGGAAGGTAAAGCGGC
GACGGCGGCTTCAGGAGCTGTGGGTTCCCCGGCTGGGAGCTGGGGGCTCGCGGGGAAATCATTTACTA
TGTCACAGTAGAACAAATATGTGAAACTTCCTGCCAATTTGTGAACAACTTGCTCAGAAGTTCTTCGA
TGCCATGTTCTGTGGCTTGCATCAAAAAAGGAGTTTGTCTTCATGAAGATTCCTAACATTGGTAATGT
GATGAATAAATTTGAGATCCTTGGGGTTGTAGGTGAAG

>Cricetulus_griseus
Ccri
GCCCCGGCAAGAGGGCGGGGCCGGAGGTTTCGATTACTTGTCTCTGCCGCTAGGGAAGGTAAAGCGGT
GAGGGCGGCCGCGGGAGTGGAGGCTTGCCCACTAGAGGCGGGGGACTCGGCAGGAGATTCACTTATTA
CTTCACCATTGCACAAATGTGAGAGTTCCTTGCCAAGCATTATTCGTCAGGACATTTTTTTCTGCTTA
CTGAATAACTTTTTTCTAAAGATCCTTTTTTGATGCCATGTTTTGTGGCTTACATCAAAAGAGGAGTT
TGTCTTCATGAAGATTCCTAACATTGGTAATGTGATGAATAAATTTGAGATCCTTGGGGTTGTAGGTG
AAG

>Capra_hircus
Chir
GCCTGAGCGAGAGGGCGGGGTCGAGGGTTTCGATTAGTTGTCGCTGCCGCTGGGGAAGGTAAAGCGGT
GACGGCGGCCTCAGGAGCTGTGGGGTCCCCGGCTGGAGGTGGGGAACTCGGCGGGGAAATCATTTAAT
AATTCACATTAGGACAAATATGTGAAAGTTCCTGCCAACCTGTGAATATTTCTTCCTTCGGACATTTT
TTGTTCTACTTACTGTACAACTTTCTTAGAAGTTCTTCTTTGATGCCATGTTCTGTGGCTTGCATCTA
AAGAGGAGTTTGTCTTCATGAAGATTCCTAACATTGGTAATGTGATGAATAAATTTGAGATCCTTGGG
GTTGTAGGTGAAG

>Panthera_pardus
Ppar

GCCTCTGCGAGAGGGCGGGGCCAGAGGTTTCCATTAGTTGTCCCTGCCACTGGGAAAGGTAAAGCGGC
GACGGCGGCCTCAGGAGCTGTGGGGTCCCCGGCTGGAGGTGGGAGACTCGGCGGGGGAATCATTTAAT
ACTTCACATTACAACAAAATGTGAAAGTTCCTGCCAACCTGTGAGAATTTCTTCCTTCAACATTTTCT
ATTTACTGCACAACTTTCTCAGTTCTTTTTTGATGCCATGTTTTGTGGCTTGCATCAAAAGAGGAGTT
TGTCTTCATGAAGATTCCTAACATTGGTAATGTGATGAATAAATTTGAGATCCTTGGGGTTGTAGGTG
AAG

>Heterocephalus_glaber
Hgla
GCTCCTGCGAGAAGGCGGGGCCGGAGGTTTCGATTAGTTGTCTCTGCCTCTGGGGAAGGTATATCGGC
GACGGCGGCTTCGGGAGCTGTGGGGTCCCGGGCTGGGGGACTCGGCGGGGACTCATTTAATATTTCAG
TATTAAAAGGAGTATGTGAAAATTCCTGCCAACCATTGAGAATTTCTTCCTTTGGACATTTTTTCTTC
TACTTACTACATAACTTTCTTGGAAGTTCCTTTTTTGGTGCCATGTTTTGTGGCTTACATCAAAGGAG
GAGTTTGTCTTCATGAAGATTCCTAACATTGGTAATGTGATGAATAAATTTGAGATCCTTGGGGTTGT
AGGTGAAG

>Suricata_suricatta
Ssur
GCCTCTGCGAGAGGGCGGGGCCGGAGGTTTCCATTAGTTGTCCCTGCCACTGGGAAAGGTAAAGCGGC
GACGGCGGCCTCAGGAGCTGTGGGGTCCCCGGCTGGAGGTGGGGGACTCGGCGGGGGGATCATTTAAT
ACTTTACATTACAACAAAAATGTGACAGTTCCTGCCCACCTGTGAGAATTTCTTCCTTCGACATTTTC
TACTTACTGCACAACTTTCTCAGAAGTTCTTTTTTGATGCCATGTTTTGTGGCTTGCATCAAAAGAGG
AGTTTGTCTTCATGAAGATTCCTAACATTGGTAATGTGATGAATAAATTTGAGATCCTTGGGGTTGTA
GGTGAAG

>Theropithecus_gelada
Tgel
GCTTCAGCTAGAGGGCGGGGCCGGAGGTTTCGATTAGTTGTCTCTGCCGCTGGGGAAGGTAAAGCGGC
GACGGCGTCCTCAGGAGCTGTGGGGTCCCCTGCTAGAAGTGGGGGACTCGGCGGGGGAATTGTTTAAT
ACTTCATGATTGCAACAAGTATGTGAAAGTTCCCACCAGCCAGTGAGAATTTCTTCCTTCAGAAGGTT
TTTGGTCTTACCGCGCAGCTTTCTGAGTTCTTTTGGTGCCATGTTTTGTGGCTTGCATCAAAAGAGGA
GTTTGTCTTCATGAAGATTCCTAACATTGGTAATGTGATGAATAAATTTGAGATCCTTGGGGTTGTAG
GTGAAG

>Eptesicus_fuscus
Efus
GCTACTGCGAGAGGGCGGGGCTGGAGGTTTCGATTAGTTGTCGCTGCCGCTGGGGAAGGTAAAGCGGC
GACCGTGGCCTCAGGAGCTCTTGGGTCCCCGGCTGGAGGTGGGGGACTTGCCGGGGAATTATTTAATA
CTTCACATTAGAAGAAATATGTGAAAGTTCCTGCCAACCTGTGAGAATTTCTTCCTTCGGCCATTTTT
TTGTTCTGCTTACTAGACAACTTTCTCAGAAGTTCTTTTTTGATGCCATGTTTTGTGGCTCGCATCAA
AAGAGGAGTTTGTCTTCATGAAGATTCCTAACATTGGTAATGTGATGAATAAATTTGAGATCCTTGGG
GTTGTAGGTGAAG

>Mus_pahari
Mpah
GCTCCGGCGAGAGGGCGGGGCCGGAGGTTTCGATTACTTGTCGCTGCCGCTAGGGAAGGTAAAGCGGT
GACGGCGGCCGCGAGCGTGGAGGCTTCCCCGATAGAGGTGGGGGACTCGGCAGGGGACTCATTTAATA
CTGCACAGTTGATCAAATGTATGAAGGTTCCTTGCCAACCATCGAGAATTTCTTCGTTGGGACATTTT
TCCCCTGTTTACTAAATAACTTGTTTAGAAGTGTCTTTTTTGGTGCCATGTTCTGTGGCTCGCATCAA

AAGAGGAGTTTGTCTTCATGAAGATTCCTAACATTGGTAATGTGATGAATAAATTTGAGATCCTTGGG
GTTGTCGGTGAAG

>Felis_catus
Fcat
GCCTCTGCGAGAGGGCGGGGCCGGAGGTTTCCATTAGTTGTCCCTGCCACTGGGAAAGGTAAAGCGGC
GACGGCGGCCTCAGGAGCTGTGGGGTCCCCGGCTGGAGGTGGGAGACTCGGCGGGGGAATCATTTAAT
ACTTCACATTACAACAAAATGTGAAAGTTCCTGCCAACCTGTGAGAATTTCTTCCTTCAACATTTTCT
ATTTACTGCACAACTTTCTCAGTTCTTTTTTGATGCCATGTTTTGTGGCTTGCATCAAAAGAGGAGTT
TGTCTTCATGAAGATTCCTAACATTGGTAATGTGATGAATAAATTTGAGATCCTTGGGGTTGTAGGTG
AAG

>Peromyscus_leucopus
Pleu
GCTCCGGCAAGAGGGCGGGGCCGGAGGTTTCGATTACTTGTCGCTGCCGCTAGGGAAGGTAAAGCGGT
GACGGCGGCCGCGGGAGTGGAGGCTTCCCCACTAGAGGTGGGGGGACTCGGCAGGGAACTCACCATTG
AACAAATGTATGAAAGTTCCTTGCCAAGCATTCTTCATTGGGACATTTGTTTTCCTGCTTACTGAATA
ACTTTTTCAAAAGTTCCTTTTTTGGTGCCATGTTCTGTGGCTTACATCAAAAGAGGAGTTTGTCTTCA
TGAAGATTCCTAACATTGGTAATGTGATGAATAAATTTGAGATCCTTGGGGTTGTAGGTGAAG

>Rattus_norvegicus
Rnor
GCTCCGGCGAGAGGGCGGGGCCGGAGGTTTCGATTACTTGTCGCTACCGCTAGGGAGAAGGTAAAGCG
GTGACGGCGGCCGCCGGAGTGGAGGCTTCCCCGGTAGAGGTGGGGGACTCAGTAGGGGACTCATTTAA
TACTTCACAGTTGAACAAATGTATGAACGTTCCTTGCCAACCATTGAGAATTTCTTCGTTGGGACATT
TTTTTCCTGCATACTGAATAACTGGTTTAGAAGTCCCTTTTTTGGTGCCATGTTCTGTGGCTTGAATC
AAAAGAGGAGTTTGTCTTCATGAAGATTCCTAACATTGGTAATGTGATGAATAAATTTGAGATCCTTG
GGGTTGTTGGTGAAG

>Vombatus_ursinus
Vurs
GCCCGGTCGGGAGGGCGGGGCCGGAGGATTCGATTAGTTGCCAGTGCCGCTGTGGTAGAGAAAACAGT
GACGGCTGCGGCGGCGGTGGCGCTGCGAGTCCCCGCTTAGAGCCGGGACAGTGGGGGGGAATCATTTT
ATACTCCAGAATTAGGACAAGTATGTGAGAATTCCCATCGGCCAGTGTGGATTTCTTTCTTCAAGCTC
TCTTCCAATACAACCAGCTATATAAGTTTCTCAAATAACCCTTCTTTGTGCCATGTTCTGTGGCTTGC
ATCAAAAGAGGAATTTGTCTTCATGAAGATTCCTAACATTGGTAATGTGATGAATAAATTTGAGATCC
TTGGGGTTGTAGGTGAAG

>Marmota_flaviventris
Mfla
TCTTCTGCGAGAGGGCGGGGCCGGAGGTTTCGATTAGTTGTCCCTGCCGCTGGGGAAGGTAAAGCGTC
GACGGCGGCTTCTGGAGCAGTGGGGTCCCCGGCTGGAGGTGGGGACTCAGCGGGGAACTCATTTAATG
CTTCATTATTAGAACAAATATGTGAAAATTCCTGTCAACTGTTGAGAATTTTTTCCTTTGGATATTGT
TTCCTCCACTTTCTGCATAAATTTCTTAGAAATTTTTTTTTGGTGCCATGTTCTGTGGCTTACATCA
AAAGAGGAGTTTGTCTTCATGAAGATTCCTAACATTGGTAATGTGATGAATAAATTTGAGATCCTTGG
GGTTGTAGGTGAAG

>Delphinapterus_leucas

Dleu
GCCTGAGAGAGAGGGCGGGGTCGAAGGTTTCGATTAGTTGTCGCCGCCGCTGGGGAAGGTAAAGCGGC
GACGGCGGCCTCGGGAGCTGTGGGACCCTGGCTGGAGGTGGGGATCTCGGCGGGGGAATCATTTAATA
CTTCACATTAGGACAAATATGTGAAAGTTCCTGCCAACCTGTGAGAATTTCTTCCTTTGGACATTTTT
TGTTCTACTTACTGTACAACTTTCTTAGAAGCTCTTTTTTGATGCCATGTTCTGTGGCTTGCATCAAA
AGAGGAGTTTGTCTTCATGAAGATTCCTAACATTGGTAATGTGATGAATAAATTTGAGATCCTTGGGG
TTGTAGGTGAAG

>Pan_troglodytes
Ptro
GCTTCTGCTAGAGGGCGGGGCCGGAGGTTTCGATTAGTTGTCTCTGCCGCTGGGGAAGGTAAAGCGGC
GACGGCGTCCTCAGGAGCTGTGGGGTCCCCTGCTAGAAGTGGGGGACTCGGCGGGGGAGTCATTTAAT
ACTTCATGATTAGAACAAATATGTGAAAGTTCCCACCAACCAGTGAGAATTTCTTCCTTCAGACGGTT
TTGGATCTTACTGCACAGCTTTCTGAGAAGTTCTTTTGGTGCCATGTTTTGTGGCTTGCATCAAAAGA
GGAGTTTGTCTTCATGAAGATTCCTAACATTGGTAATGTGATGAATAAATTTGAGATCCTTGGGGTTG
TAGGTGAAG

>Bos_indicus
Bind
GCCTGAGCGAGAGGGCGGGGTCGAGGGCTTCGATTAGTTGTCGCTGCCGCTGGGGAAGGTAAAGCGGT
GACGGCGGCCTCAGGAGCTGTGGGGTCCCCGGCTGGAGGTGGGGAACTCGGCGGGGAAATCATTTAAT
AATTCACATTAGGACAAATATGTGAAAGTTCCTGCCAACCTGTGAATATTTCTTCCTTCGGACATTTT
TTGTTCTACTTACTGTACAACTTTCTTAGAAGTTCTTTTTTGATGCCATGTTCTGTGGCTTGCATCTA
AAGAGGAGTTTGTCTTCATGAAGATTCCTAACATTGGTAATGTGATGAATAAATTTGAGATCCTTGGG
GTTGTAGGTGAAG

>Pongo_abelii
Pabe
GCTTCTGCTAGAGGGCGGGGCCGGAGGTTTCGATTAGTTGTCTCTGCCGCTGGGGAAGGTAAAGCGGC
GACGGCGTTCTCAGGAGCTGTGGGGTCCCCTGCTAGAAGTGGGGGACTCGGCGGGGGAGTCATTTAAT
ACTTCGTGATTAGAACAAATATGTGAAAGTTCCCACCAACCAGTGAGAATTTTTTCCTTCAGACGGTT
TTGGATCTTACGACACAGCTTTCTGAGAAGTTCTTTTGGTGCCATGTTTTGTGGCTTGCATCAAAAGA
GGAGTTTGTCTTCATGAAGATTCCTAACATTGGTAATGTGATGAATAAATTTGAGATCCTTGGGGTTG
TAGGTGAAG

>Bos_taurus
Btau
GCCTGAGCGAGAGGGCGGGGTCGAGGGCTTCGATTAGTTGTCGCTGCTGCTGGGGAAGGTAAAGCGGT
GACGGCGGCCTCAGGAGCTGTGGGGTCCCCGGCTGGAGGTGGGGAACTCGGCGGGGAAATCATTTAAT
AATTCACATTAGGACAAATATGTGAAAGTTCCTGCCAACCTGTGAATATTTCTTCCTTCGGACATTTT
TTGTTCTACTTACTGTACAACTTTCTTAGAAGTTCTTTTTTGATGCCATGTTCTGTGGCTTGCATCTA
AAGAGGAGTTTGTCTTCATGAAGATTCCTAACATTGGTAATGTGATGAATAAATTTGAGATCCTTGGG
GTTGTAGGTGAAG

>Enhydra_lutris
Elut
GCTTCGGCGAGAGGGCGGGGCCGGAGGTTTCCATTAGTTGTCCGTGCCACTGGGAAAGGTAAATCGGC
GACGGCGGCTTCAGGAGCTGTGGGGTCCCCGGCTGGAGGTGGGGGACTCGGCTGGGGAATAATTTAAT
TCTTCACATTAGAACAAATATGTGAAAGTCCCTGCCAACCTGTGAGAATTTCTTCCTTTGACATTTTG

ATTCTACTTTACAACGTTCTCTGAAGTTCTTTCTTGATGCCATGTTCTGTGGCTTGCATCAAAAGAGG
AGTTTGTCTTCATGAAGATTCCTAACATTGGTAATGTGATGAATAAATTTGAGATCCTTGGGGTTGTA
GGTGAAG

>Dasypus_novemcinctus
Dnov
GCCTCTTCGAGAGGGCGGGGCCGGAGGTTTCGATTAGTGGTTGTTGCCGCTGGGGACGGTGAAGCGGC
GACGGCGGCGGCCTCGGGAGCTGTGGGGTCCCGGGCCGGAGGTGGGGGATTCGGCGGGGGAATCATTT
ATTGCTTGACTATTACAACAAATATGTGAAAGTTCCTGTCAACCAGTGAGAGTTTCCTTCTTCAGACA
TTCTTTGTTCTGCTTACTGCACAACTTTCTCCAAAGATCGTTTTTGATGCCATGTTCAGTGGCTTGCA
TCAGCAGAGGAATTTGTCTTCATGAAGATTCCTAATATTGGTAATGTGATGAATAAATTTGAGATCCT
TGGGGTTGTAGGTGAAG

>Mus_caroli
Mcar
GCTCCGGCGAGAGGGCGGGGCCGGAGGTTTCGATTACTTGTCGCTGCCGCTAGGGAAGGTAAAGCGGT
GACGGCGGCCGCGAGAGTGGAGGCTTCCCCGATAGAGGTGGGGGACTCGGCTGGGGGCTCAGTTAATA
CTGCACAGTTGAACAAATGTATGAAGGTTCCTTGCCAACCATTGAGAATTTCTTCGTTGGGACATTAT
TTCCCTGCTTACTGAATAACTTGTTTAGAAGTTTCTTTGTTGGTGCCATGTTCTGTGGCTCGCATCAA
AAGAGGAGTTTGTCTTCATGAAGATTCCTAACATTGGTAATGTGATGAATAAATTTGAGATCCTTGGG
GTTGTCGGTGAAG

>Nomascus_leucogenys
Nleu
GCTTCTGCTAGAGGGCGGGGCCGGAGGTTTCGATTAGTTGTTTCTGCCGCTGGGGAAGGTAAAGCGGC
GACGGCGTCTTCAGGAGCTGTGGGGTCCCCTGCTAGAAGTGGGGGACTCGGCGGGGGAGTCATTTAAT
ACTTCATGATTAGAACAAATATGTGAAAGTTCCCACCAACCAGTGAGAATTTCTTCCTTCAGACGGTT
TTGGTTCTTACTGCACAGCTTTCTGAGAAGTTCTTTTGGTGCCATGTTTTGTGGCTTGCATCAAAAGA
AGAGTTTGTCTTCATGAAGATTCCTAACATTGGTAATGTGATGAATAAATTTGAGATCCTTGGGGTTG
TAGGTGAAG

>Papio_anubis
Panu
GCTTCTGCTAGAGGGCGGGGCCGGAGGTTTCGATTAGTTGTCTCTGCCGCTGGGGAAGGTAAAGCGGC
GACGGCGTCCTCAGGAGCTGTGGGGTCCCCTGCTAGAAGTGGGGGACTCGGCGGGGGAATTGTTTAAT
ACTTCATGATTGCAACAAGTATGTGAAAGTTCCCACCAGCCAGTGAAAATTTCTTCCTTCAGAAGGTT
TTTGGTCTTACCGCGCAGCTTTCTGAGTTCTTTTGGTGCCATGTTTTGTGGCTTGCATCAAAAGAGGA
GTTTGTCTTCATGAAGATTCCTAACATTGGTAATGTGATGAATAAATTTGAGATCCTTGGGGTTGTAG
GTGAAG

>Pteropus_alecto
Pale
GCTTTTGCGAGAGGGCGGGGCCAGAGGTTTCGTTTAGTTGTCCCTGCAGCTGGGGAAGGTAAAGCGGC
GACGGCGGCTTCAGGAGCTGTGGGTTCCCCGGCTGGGAGCTGGGGGCTCGCGGGGAAATCATTTACTA
TGTCACAGTAGAACAAATATGTGAAACTTCCTGCCAATTTGTGAACAACTTGCTCAGAAGTTCTTCGA
TGCCATGTTCTGTGGCTTGCATCAAAAAAGGAGTTTGTCTTCATGAAGATTCCTAACATTGGTAATGT
GATGAATAAATTTGAGATCCTTGGGGTTGTAGGTGAAG

>Otolemur_garnettii
Ogar
GCGTCTGTTAGAGGGCGGGGCCGGAGGTTTCGGTTAGTTGTCTGTGCGGCTGGGGAAGGTACAGAAGC
GACGGCGGCCTCAGGAGCTGTGGGGTCCCCTGCTGGAGGCGGGGGACTCAGCTGGGGTTTAATACGTC
ACTATTGAACAAATATGTGAAAATTCCTGCCAGCGAGTGACAATTTCTTCCTTTGGACATTTTTTGTT
CTTCTTACTGCACAACTGTTTTAGAAATCCCTTTTTTGGTGCCATGTTCGGTGGCGTACATCAAAAGA
GGAGTTCGTCTTCATGAAGATTCCTAACATTGGTAATGTGATGAATAAATTTGAGATTCTTGGGGTTG
TAGGTGAAG

>Cavia_porcellus
Cpor
GCTCCTGCGAGAGGGCGGGGCCGGAGGTTTCGATTAGTTGTCGCTGCCGCTGGGGAAGGTAAAGCGGA
GACGGCGGCCTCAGGAGCTGAGGGGGTCCCCGGCAGGAGGTGGGGGACTCGACGGGGGACTCATTTAA
TATTTCACTATTAAAAGGAATATGTGAAAATTTCTGCCAACCATTGAGAATTTCTTCCTTTGGACATT
TTTCTTCTACTTTATATATAATTTTCTCAGAAGTTCCTTTTTTGGTGCCATGTTTTGTGGCTTACATC
AAAAGAGGAGTTTGTCTTCATGAAGATTCCTAACATTGGTAATGTGATGAATAAATTTGAGATCCTTG
GGGTTGTAGGTGAAG

>Bubalus_bubalis
Bbub
GCCTGAGCGATAGGGCGGGGTCGAGGGTTTCGATTAGTTGTCGCTGCCGCTGGGGAAGGTAAAGCGGT
GACGGCGGCCTCGGGAGCTGTGGGGTCCCCGGCCGGAGGTGGGGAACTCGGCGGGGAAATCATTTAAT
AATTCACATTAGGACAAATATGTGAAAGTTCCTGCCAACCTGTGAATATTTCTTCCTTCGGACATTTT
TTGTTCTACTTACTGTACAACTTTCTTAGAAGTTCTTTTTTGATGCCATGTTCTGTGGCTTACATCTA
AAGAGGAGTTTGTCTTCATGAAGATTCCTAACATTGGTAATGTGATGAATAAATTTGAGATCCTTGGG
GTTGTAGGTGAAG

>Zalophus_californianus
Zcal
GCCTCTGCGAGAGGGCGGGGCCGGAGGTTTCCATTAGTTGTCCCTGCCACTGGGAAAGGTAAATCGGC
GACGGCGGCCTCAGGAGCTGTGGGGTCCCCGGCTGGAGGTGGGGGACTCGGCTGGGGAATCATTTAAT
ACTTCACATTAGAACAAATATGTGAAAGTCCCTGCCAACCTGTGAGAATTTCTTCCTTCGACATTTTG
GTTCTACTTCACAACTTTCTTAGAAGTTCTTTCTTGATGCCATGTTCTGTGGCTTGCATCAAAAGAGG
AGTTTGTCTTCATGAAGATTCCTAACATTGGTAATGTGATGAATAAATTTGAGATCCTTGGGGTTGTA
GGTGAAG

>Mesocricetus_auratus
Maur
GCCCCGGCGAGAGGGCGGGGCCGGAGGTTTCGATTACTTGTCTCTGCCGCTAGGGAAGGTAAAGCGGT
GAGGGCGGTCGCGGGAGTGGAGGCTTCCCCAGTAGAGGCGGGGGACTCGGCAGGGGACTCATTTAATG
CTTCACCATTGAACAAATGTATGAGAGTTCCTTGCCAAGCATTCTTCGTCGGGACATTTTTCTTTCTG
TTTACTGAATAACTTTTTCAAAAGTTCCTTTTTTGATGCCATGTTTTGTGGCTTACATCAAAAGAGGA
GTTTGTCTTCATGAAGATTCCTAACATTGGTAATGTGATGAATAAATTTGAGATCCTTGGGGTTGTAG
GTGAAG

>Hipposideros_armiger
Harm
CCCTGATGCGAGAGGGCGGGGCCGGAGGTTTCGATTAGTTATTACTGCCGCTGGGGAAGGTAAAGCGG
CGACGGCGGGCTCTGGAGCTGTGGAGTCCCTGGCTGGAGGTGGGGGACTCGTGGGGGAGTCATTTAAT

ACTTCACATTAGAACAAATATGTGAAAGTTCCTGCCAACCTGTGAACAACTTTCTCAGAAGTTCTTTT
TTGATGCCATGTTCTGTGGCTTGCATCAAAAGAGGAGTTTGTCTTCATGAAGATTCCTAACATTGGTA
ATGTGATGAATAAATTTGAGATCCTTGGGGTTGTAGGTGAAG

>Cebus_imitator
Cimi
GCTTCTGTTAGAGGGCGGGGCCGGAGGTTTCGGTTAGTTGTCTCTGCCGTTGGGGAAGGTAAAGCGGA
GACGGCGTCCTCAGGAGCTGTGGGGTCCCCTGCTAGAAGTGGGGGACTCGGCAGGGAAATCATTTAAT
ACTTGATGATTAGAACAGATATGTGAAAGCTCCCACCAACCAGTGAGAATTTCTTCCTTCAGAAGGGT
TTTTGTTCTTAACTGCACAGCTTTCTAAGAAGTTCTTTTGGTGCCATGTTTTATGGCTTGCATCAAAA
GAGGAGTTTGTCTTCATGAAGATTCCTAACATTGGTAATGTGATGAATAAATTTGAGATCCTTGGGGT
TGTAGGTGAAG

>Microcebus_murinus
Mmur2
GCTTCTTCGAGAGGGCGGGGCCGGAGGTTCCGATTAGTTGTCTGTGCCGCTGGGGAAGGTAAAGCAGC
GACGGCGGCCTCAGGAGCTGTACGATCTCCGGCTGGCGGAGTGAGACTCAGCGGGGGAGTCATTTAAT
ACTTCACTACTAGAACAAATATGTGAAAGTTCCTGCCAAACAGTGAGAATTTCTTCCTTCGGACATTT
TTTGTTCTACTTACTGCACAGCTTTCTCAGTAGTCCCTTTTTTGGTGCCATGTTCTGTGGCTTGCATC
AAAAGAGGAGTTTGTCTTCATGAAGATTCCTAACATTGGTAATGTGATGAATAAATTTGAGATCCTTG
GGGTTGTAGGTGAAG

>Rousettus_aegyptiacus
Raeg
GCTTTTGCGAGAGGGCGGGGCCAGAGGTTTCGATTAGTTGTTCCTGCAGCTGGGGAAGGTAAAGCGGC
AACGGCGGCTTCAGGAGCTGTGGGTTCCCGGGCTGGAAGCTGGGGGCTCGTGGGGAAATCATTTACTA
TGTCACAGTAGAACAAATATGTGAAACTTCCTGCCAGTTTGTGAACAACTTGCTCAGAAGTTCTTTGA
TGCCATGTTCTGTGGCTTGCATCAAAAAAGGAGTTTGTCTTCATGAAGATTCCTAACATTGGTAATGT
GATGAATAAATTTGAGATCCTTGGGGTTGTAGGTGAAG

>Canis_lupus_familiaris
Clupf
GTTCGCCTCTGCGAGAGGGCGGGGCCGGAGGTTTCCATTAGTTTCCCTGCCACTGGAAAGTAAAGCGG
CGACGGCGGCCTCAGGAGCTGGGGTCCCCGGCTGGAGGTGGGGGACTCAGCGGGGGAATCATTTCATA
CTTCACATTTGAATAAATATGTGAAAATCCCTGTCAACCTGTGAGAATTTCTTCTTTCGACATTTTTG
TTCTACTTCACAACTTTCTCAGAAGTTCTTTCTTGATGCCATGTTCTGTGGCTTGCATCAAAAGAGGA
GTTTGTCTTCATGAAGATTCCTAACATTGGTAATGTGATGAATAAATTTGAGATCCTTGGGGTTGTAG
GTGAAG

>Rhinopithecus_roxellana
Rrox
GCTTCTGCTAGAGGGCGGGGCCGGAGGTTTCGATTAGTTGTCTCTGCCGCTGGGGAAGGTAAAGCGGC
GACGGCGTCCTCAGGAGCTGTGGGGTCCCCTGCTAGAAGTGGGGGACTCGGCGGGGGAATCGTTTAAT
ACTTCATGATTGCAACAAGTATGTGAAAGTTCCCACCAGCCAGTGAGAATTTCTTCTTTCAGAAGGTT
TTTGTTCTTACCGCGCAGCTTTCTGAGTTCTTTTGGTGCCATGTTTTGTGGCTTGCATCAAAAGAGGA
GTTTGTCTTCATGAAGATTCCTAACATTGGTAATGTGATGAATAAATTTGAGATCCTTGGGGTTGTAG
GTGAAG

>Piliocolobus_tephrosceles
Ptep
GCTTCTGCTAGAGGGCGGGGCCGGAGGTTTCGATTAGTTGTCTCTGCCGCTGGGGAAGGTAAAGCGGC
GACGGCGTCCTCAGGAGCTGTGGGGTCCCCTGCTAGAAGTGGGGGACTCGGCGGGGGAATCCTTTAAT
ACTTCATGATTGCAACAAGTATGTGAAAGTTCCCACCAGCCAGTGAGAATTTCTTCCTTCAGAAGGTT
TTTGTTCTTACCGCGCAGCTTTCTGAGTTCTTTTGGTGCCATGTTTTGTGGCTTGCATCAAAAGAGGA
GTTTGTCTTCATGAAGATTCCTAACATTGGTAATGTGATGAATAAATTTGAGATCCTTGGGGTTGTAG
GTGAAG

>Gorilla_gorilla
Ggor
GCTTCTGCTAGAGGGCGGGGCCGGAGGTTTCGATTAGTTGTCTCTGCCGCTGGGGAAGGTAAAGCGGC
GACGGCGTCCTCAGGAGCTGTGGGGTCTCCTGCTAGAAGTGGGGGACTCGGCGGGGGAGTCATTTAAT
ACTTCATGATTAGAACAAATATGTGAAAGTTCCCACCAACCAGTGAGAATTTCTTCCTTCAGACGGTT
TTGGATCTTACTGCACAGCTTTCTGAGAAGTTCTTTTGGTGCCATGTTTTGTGGCTTGCATCAAAAGA
GGAGTTTGTCTTCATGAAGATTCCTAACATTGGTAATGTGATGAATAAATTTGAGATCCTTGGGGTTG
TAGGTGAAG

>Ursus_arctos_horribilis
Uarc
GCCTTTGCGAGAGGGCGGGGCCGGAGGTTTTCATTAGTTGTCCCTGCCACTGGGAAAGGTAAATCGGA
GACGGCGGCCTCAGGAGCTGTGGGGTCCCCGGCTGGAGGTGGGGGACTTGGCTAGGGAATCATTTAAT
ACTTCACATTAGAACAAATATGTGAAAGTCCCTGCCAGCCTGTGAGAATTTCTTCCTTCGACATTTTG
GTTTTACTTCACAACTGTCTCTGAAGTTCTTTCTTGATGCCATGTTCTGTGGCTCGCATCAAAAGAGG
AGTTTGTCTTCATGAAGATTCCTAACATTGGTAATGTGATGAATAAATTTGAGATCCTTGGGGTTGTA
GGTGAAG

>Myotis_lucifugus
Mluc
GCTACTGCGAGAGGGCGGGGCTGGAGGTTTCGATTAGTTGTCGCTGCCGCTGGGGAAGGTAAAGCGGC
GCCCGTGGCCTCAGGAGCTCTTGGGTCTCCGGCTGGAGGTGGGGGACTTGTCGGGAATCATTTAATAC
TTCACATTAGAAGAAATATGTGAAAGTTCCTGCCAACCTGTGAGAATTTCTTCCTTCGGCCATTTTTT
TGTTCTACTTACTAGACAACTTTCTCAGAAGTTCTTTTTTGATGCCATGTTTTGTGGCTCGCATCAAA
AGAGGAGTTTGTCTTCATGAAGATTCCTAACATTGGTAATGTGATGAATAAATTTGAGATCCTTGGGG
TTGTAGGTGAA

>Trichosurus_vulpecula
Tvul
GCCCGGTCGGGAGGGCGGGGCCGGAGAATTCTATTAGTTGCCACTGCCACTGGGGTAGAGAAAACAGC
GTCGGCTGCGGCGGCTGTAGCGGTGGGAGCCCCCGCTTAGAGCCGGCATAGAGTGGGGGGGAATCATT
TTATACTTCAGAATTAGGACAAATATGTGAGAATTCCCATCAGCCAGCGTGGATTTCTTTCTTCAAAC
TCTTCCAATACAACCAGCTACATAACTTTCTCAAATAACCCTTCTTTGTGCCATGTTCTGTGGCTTGC
ATCAAAAGAGGAATTTGTCTTCATGAAGATTCCTAACATTGGTAATGTGATGAATAAATTTGAGATCC
TTGGGGTTGTAGGTGAAG
