# Supplementary material for: Complex CDKL5 translational regulation and its potential role in CDKL5 deficiency disorder
Source: Front Cell Neurosci. 2023 Oct 30;17:1231493. doi: 10.3389/fncel.2023.1231493 (PMC10642286; doi:10.3389/fncel.2023.1231493)
Supplement: Supplementary file 2 [file Data_Sheet_2.pdf]

Supplementary Material

1 SUPPLEMENTARY DATA

1.1 Supplementary 1: List of the exonic sequences composing the 5'UTR variants of CDKL5

- **201a**:
ACCTCAGGAC TCTTTAGAGC TCTGAGTGAG TGGGGTGGGG GAGTTGGAGG AGGAAGACCC TACAGGAAGT TGAG
- **201b**:
CTGTACTCTC CAACAGGCGA GCACATGGTC GTCTCCTCTC TCCCTTCAG
- **209**:
TGGCTGCCCG CGAGGGTGGG CTAGCGACCG GGCGCCGGCC CCGCGGCTCC GCAACCTCCC CCGGACAAGT TTCAGGCGAT GCTGTGAAGT TCACGACTGG CATAGTGCGG GGAAGAAGAA TGGCCCCTTC TCAGACCTTC ATACCTTTTG GACCCTCATA GAAATCGGAT TCTGGGATTT GGAACCGTCT CCCAGAACGA ACAG
- **202**:
GAGGGCGGGG CCGGAGGTTT CGATTAGTTG TCTCTGCCGC TGGGGAAGGT AAAGCGGCGA CGGCGTCCTC AGGAGCTGTG GGGTCCCCTG CTAGAAGTGG GGGACTCGGC GGG
- **203/HecF**:
AATTTCTGTG ACCCCCAGTG GTTTCACACT ATCTTTTCAG CAAGCCACAT TGGATTTCAC GCATTTACTG ACTCTTCTAC CCGTTCTTTT TGGTGTCCAG CTGTACTCTC CAACAG
- **207**:
GAGTTGGAGG AGGAAGACCC TACAGGAAGT TGAGGTATCA TATCCTCAAT TTCTGTGACC CCCAGTGGTT TCACACTATC TTTTCAGCAA GCCACATTGG ATTTCACGCA TTTACTGACT CTTCTACCCG TTCTTTTTGG TGTCCAGCTG TACTCTCCAA CAGGCGAGCA CATGGTCGTC TCCTCTCTCC CTTCAG
- **204a**:
GGGGGAGTTG GAGGAGGAAG ACCCTACAGG AAGTTGAG
- **204b**:
CTGTACTCTC CAACAGGCGA GCACATGGTC GTCTCCTCTC TCCCTTCAG
- **204c**:
GCTGCTCATC TTCAAGGGGA AAACATGGCT ACTGGATTTC CTCTGTTCTA AG
- **205/213/HecA**:
AGTTGTCTCT GCCGCTGGGG AAGGTAAAGC GGCGACGGCG TCCTCAGGAG CTGTGGGGTC CCCTGCTAGA AGTGGGGGAC TCGGCGGG
- **211**:
TTGTTTGAAC GTAATGGTTA TATAGTTAAT GTAAAAGATT ACACAAATTA ATTTGTCTGA GAGCGTTTGT CAGAAACAGG AGACAGAGAT GTGTATAGAA CAGGATTATC GTTTTAGGTA ACATCTGCTG GAACATTTCC AGCTTGTTTC TTTCTTGCTG TAGGGTTACA AAAAAGACAT CTTCACCGAG GCTATTCGGA ATTACAT

- **208**:
GTGAGTGGGG TGGGGGAGTT GGAGGAGGAA GACCCTACAG GAAGTTGAGG TATCATATCC TCAATTTCTG TGACCCCCAG TGGTTTCACA CTATCTTTTC AGCAAGCCAC ATTGGATTTC ACGCATTTAC TGACTCTTCT ACCCGTTCTT TTTGGTGTCC AGCTGTACTC TCCAACAGGC GAGCACATGG TCGTCTCCTC TCTCCCTTCA G
- **HecA1**:
TTGCGGCGAG GTAAGCCCCT TCCCGCCGGC GCCGAGGAGG GGCCTGCGCG CCCCAGCCCC CTGCCCGAGT TCGGAGGGAG CCCGAGACTC GCCAGGGGCC GTCAGTCGCG GCCCGGGCCC TTCTGACCTT GGCTGCCCGC GAGGGTGGGC TAGCGACCGG GCGCCGGCCC CGCGGCTCCG CAACCTCCCC CGGACAAGTT TCAGGCGATG CTGTGAAGTT CACGACTGGC ATAGTGCGGG GAAGAAGAAT GGCCCCTTCT CAGACCTTCA TACCTTTTGG ACCCTCATAG AAATCGGATT
- **HecB**:
TTTCTGTGCA TCTGAATTAG GTTTGTTTGA ACGTAATGGT TATATAGTTA ATGTAAAAGA TTACACAAAT TAATTTGTCT GAGAGCGTTT GTCAGAAACA GGAGACAGAG ATGTGTATAG AACAGGATTA TCGTTTTAGG TAACATCTGC TGGAACATTT CCAGCTTGTT TCTTTCTTGC TGTAGGGTTA CAAAAAAGAC ATCTTCACCG AGGCTATTCG GAATTACAT
- **HecBshort**:
GGTTACAAAA AAGACATCTT CACCGAGGCT ATTCGGAATT ACAT
- **HecC**:
GGGGGAGTTG GAGGAGGAAG ACCCTACAGG AAGTTGAG
- **HecD**:
CTGTACTCTC CAACAGGCGA GCACATGGTC GTCTCCTCTC TCCCTTCAG
- **HecCD**:
GGGGGAGTTG GAGGAGGAAG ACCCTACAGG AAGTTGAGGT ATCATATCCT CAATTTCTGT GACCCCCAGT GGTTTCACAC TATCTTTTCA GCAAGCCACA TTGGATTTCA CGCATTTACT GACTCTTCTA CCCGTTCTTT TTGGTGTCCA GCTGTACTCT CCAACAGGCG AGCACATGGT CGTCTCCTCT CTCCCTTCAG
- **HecEshort**:
TTCAAGTGAT CCTCCTCAGC CTCCCAAAGA TCTGGGATTA CAGGTGTGAA CCACTGCACC TGGCCTAGTT TTATTTGCTA ATGACAAGTC AACAGTCAGT TTAGTTGTAA TTTCGTTGTA G
- **HecE**:
GCTGGTCTGG AACTCCTGGC TTCAAGTGAT CCTCCTCAGC CTCCCAAAGA TCTGGGATTA CAGGTGTGAA CCACTGCACC TGGCCTAGTT TTATTTGCTA ATGACAAGTC AACAGTCAGT TTAGTTGTAA TTTCGTTGTA G
- **UTex2**:
GGAGTCATTT AATACTTCAT GATTAGAACA AATATGTGAA AGTTCCCACC AACCAGTGAG AATTTCTTCC TTCAGACGGT TTTGGATCTT ACTGCACAGC TTTCTGAGAA GTTCTTTTGG TGCCATGTTT TGTGGCTTGC ATCAAAAGAG GAGTTTGTCT TC

Here are reported all the nucleotide sequences of the first alternative exons of CDKL5, together with the common UTex2 sequence. Exonic sequences retrieved from the Ensembl database are reported in the first box. The sequences experimentally defined by Hector (Hector et al,. 2017) are reported in the second box with the prefix Hec.

1.2 Supplementary 2: Sequences homologous to the human CDKL5 5'UTR variant 202up

List of the 64 sequences returned by the nBLAST analysis as conducted in the paper. These sequences were used for the Consurf alignment of Fig.2. Each sequence is named with a code indicating with the first major letter the genus and the last three minors the species. Please refer to the file Supplementary2.txt.

1.3 Supplementary Tables

1.3.1 Table S1

	NetStart-1.0 Analysis			TIS Miner	
**Name**	**Pos.**	**Score**	**Pred**	**Score**	**Identity to Kozak consensus [AG]XXATGG**
UTex2	19	0.16	No	0.379	CXXATGA
	34	0.216	No	0.235	TXXATGT
	125	0.073	No	0.39	CXXATGT
202	No	No	No	No	
205	No	No	No	No	
201	99	0.112	No	0.03	CXXATGG
203	No	No	No	0.03	CXXATGG
204	63	0.094	No	0.216	CXXATGG
	112	0.26	No	0.02	CXXATGG
207	172	0.16	No	0.034	CXXATGG
208	187	0.62	Yes	0.484	CXXATGG
	312	0.051	No	0	AXXATGA
209	79	0.246	No	0.209	GXXATGC
	120	0.159	No	0.101	AXXATGG
211	14	0.159	No	0.011	AXXATGG
	29	0.18	No	0.017	AXXATGT
	89	0.589	Yes	0.061	GXXATGT
Hector_a1	208	0.17	No	0.241	GXXATGT
	249	0.73	No	0.011	AXXATGG
Hector_b1	36	0.173	No	0.019	AXXATGG
	51	0.161	No	0.017	AXXATGT
	111	0.595	Yes	0.061	GXXATGT
Hector_b	No	No	No	No	
Hector_cd	63	0.081	No	0	AXXATGA
Hector_e	81	0.053	No	0.009	CXXATGG
Hector_e1	101	0.049	No	0	AXXATGA

Table S1: The predictive analysis was performed using two distinct algorithms to detect upstream start sites in the exonic sequences of CDKL5. Among the possible start sites, NetStart 1.0 prediction returned score values slightly exceeding the threshold of 0.5 only for three of them. Predictions obtained using TIS Miner (Liu et al. 2004) returned even lower scores.

1.3.2 Table S2

ID	Lenght	MFE ΔG (kcal/mol)	MFEden	GC%
202	-101.4	-77.3888	-8.99	50.9
205/207/Hector_a	-91.6	-69.631	-9.08	49.2
Hector_a1	-170.7	-136.301	-7.58	58
209	-115.3	-105.8965	-2.63	51.1
Hector_b1	-99.2	-113.5883	3.76	37.1
211	-93.4	-106.787	3.71	37.1
Hector_b	-55.1	-55.8605	0.38	39.3
201	-88.8	-80.5848	-2.97	46.7
208_long	-148.9	-152.6685	0.74	45.7
204_short:Hector_cd	-74.5	-69.3105	-2.15	45.8
203	-68.4	-78.4295	3.71	41.7
208_short/Hector_e1	-77.5	-85.9813	2.88	42.6
Hector_e	-71.1	-80.0445	3.25	41
204_long	-94.2	-85.4578	-2.98	45.8

Table S2: All the ΔG (MFE) of the CDKL5 5'UTR variants were further analyzed through the normalization method provided by Trotta (Trotta, 2014). Percentage of GC content (GC%), number of nucleotides (NTDs), and ΔG (MFE) were reported for each sequence. MFE Ref was obtained from the Dataset S1 (Trotta, 2014) of reference sequences with the same length of our sequences of interest. The comparison of the calculated MFEden to the MFEden Ref range indicates that the ΔG (MFE) is enough negative to be characteristic of a sequence able to fold itself in an ordered structure. Variants 205 and 202 ΔG (MFE) resulted to be validated by this analysis, such as variants 204 by Ensembl (composing by Ex1 204a, 204b, 204c and UTex2), and the shorter Ref 204 (composing by Ex1 204a, 204b and UTex2).

1.3.3 Table S3

Tukey's multiple comparisons test	Summary	Adjusted P Value
UTex2 vs. 205	***	0,0009
UTex2 vs. 202up	****	<0,0001
UTex2 vs. EVA	****	<0,0001
UTex2 vs. Empty	****	<0,0001
UTex2 vs. BX1	****	<0,0001
205 vs. 202up	ns	0,1484
205 vs. EVA	ns	0,2802
205 vs. Empty	***	0,0003
205 vs. BX1	****	<0,0001
202up vs. EVA	ns	0,9991
202up vs. Empty	ns	0,0814
202up vs. BX1	****	<0,0001
EVA vs. Empty	*	0,0404
EVA vs. BX1	****	<0,0001
Empty vs. BX1	**	0,0060

Table S3: Statistical analysis (Tukey's multiple comparisons test) of the results reported in Figure 7.

1.3.4 Table S4

Tukey's multiple comparisons test	Summary	Adjusted P Value
UTex2 vs. 205	****	<0,0001
UTex2 vs. 202up	**	0,0043
UTex2 vs. EVA	ns	0,6717
205 vs. 202up	ns	0,1345
205 vs. EVA	***	0,0003
202up vs. EVA	ns	0,0516

Table S4: Statistical analysis (Tukey's multiple comparisons test) of the results reported in Figure 8.

1.4 Supplementary Figures

1.4.1 Figure S1

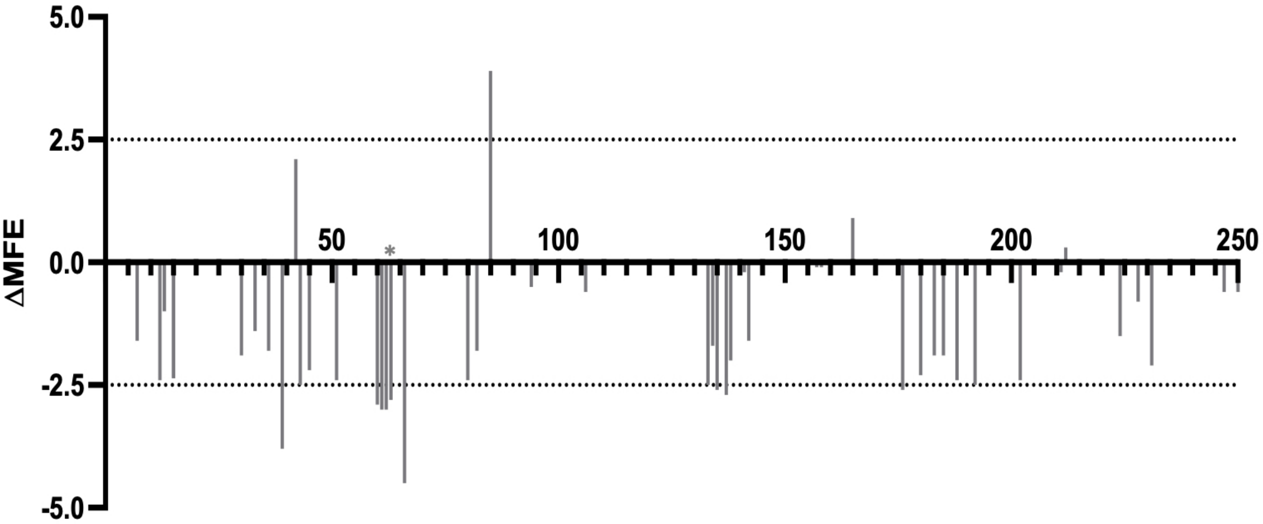

A

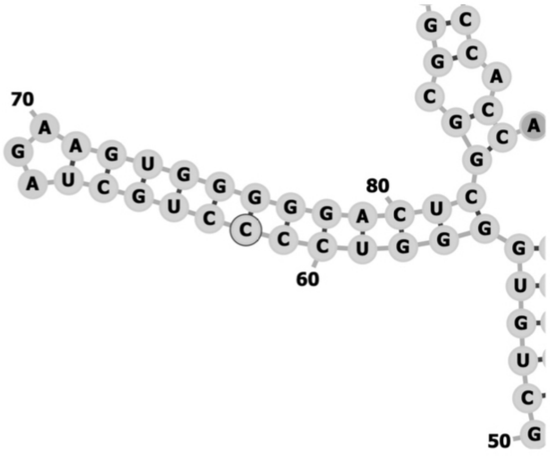

B

Figure S1: ΔMFE of the 5'UTR 205 caused by all the possible single transitions C>T. ΔMFE of the 5'UTR 205 caused by all the possible single transitions C>T. **(A)** The graph reports the variations of the difference between ΔG205 and ΔGmut (ΔMFE = ΔG205 - ΔGmut) caused by all the possible single transition C>T in the CDKL5 5'UTR 205 sequence. As expected for an ordered 5'UTR, the majority of the transition returned a negative MFE, indicating that the transition worsen the stability of the structure. This effect was rarely quantified over the ΔMFE of -2,5 kcal/mol. Noteworthy, the C tract ranging from position 60 to 63, including the position 62 (*), involved in the transition found by Evans (Evans et al., 2005), cross this threeshold, hinting their importance in the stabilization of the Watson and Crick structure, **(B)** Graphic representation of the stem-loop in which the C-tract returning the most disturbing transition lies. C62 is circled.
